# Multi-tiered analyses of honey bees that resist or succumb to parasitic mites and viruses

**DOI:** 10.1186/s12864-021-08032-z

**Published:** 2021-10-06

**Authors:** Daniel B. Weaver, Brandi L. Cantarel, Christine G. Elsik, Dawn L. Boncristiani, Jay D. Evans

**Affiliations:** 1 Genformatic, LLC, Austin, TX USA; 2 Bioinformatics Core Facility, Lyda Hill Department of Bioinformatics, UT Southwestern Medical Center, Dallas, TX USA; 3 University of Missouri, Division of Animal Sciences, Division of Plant Sciences & Technology, and Institute for Data Science and Informatics, Columbia, MO USA; 4 USDA-ARS Bee Research Laboratory, Beltsville, MD USA

**Keywords:** RNA sequencing, Host-pathogen interactions, Iflavirus, *Apis mellifera*, varroa, Pollination, Innate immunity

## Abstract

**Background:**

*Varroa destructor* mites, and the numerous viruses they vector to their honey bee hosts, are among the most serious threats to honey bee populations, causing mortality and morbidity to both the individual honey bee and colony, the negative effects of which convey to the pollination services provided by honey bees worldwide. Here we use a combination of targeted assays and deep RNA sequencing to determine host and microbial changes in resistant and susceptible honey bee lineages. We focus on three study sets. The first involves field sampling of sympatric western bees, some derived from resistant stock and some from stock susceptible to mites. The second experiment contrasts three colonies more deeply, two from susceptible stock from the southeastern U.S. and one from mite-resistant bee stock from Eastern Texas. Finally, to decouple the effects of mites from those of the viruses they vector, we experimentally expose honey bees to DWV in the laboratory, measuring viral growth and host responses.

**Results:**

We find strong differences between resistant and susceptible bees in terms of both viral loads and bee gene expression. Interestingly, lineages of bees with naturally low levels of the mite-vectored Deformed wing virus, also carried lower levels of viruses not vectored by mites. By mapping gene expression results against current ontologies and other studies, we describe the impacts of mite parasitism, as well as viruses on bee health against two genetic backgrounds. We identify numerous genes and processes seen in other studies of stress and disease in honey bee colonies, alongside novel genes and new patterns of expression.

**Conclusions:**

We provide evidence that honey bees surviving in the face of parasitic mites do so through their abilities to resist the presence of devastating viruses vectored by these mites. In all cases, the most divergence between stocks was seen when bees were exposed to live mites or viruses, suggesting that gene activation, rather than constitutive expression, is key for these interactions. By revealing responses to viral infection and mite parasitism in different lineages, our data identify candidate proteins for the evolution of mite tolerance and virus resistance.

**Supplementary Information:**

The online version contains supplementary material available at 10.1186/s12864-021-08032-z.

## Background

Parasitic mites present the single greatest threat to managed and wild honey bee (*Apis mellifera*) colonies in much of the world. The mite *Varroa destructor* strongly impacts honey bee colonies on all continents except Australia and Antarctica [1]. These mites directly impact honey bee health [2] and transmit a range of devastating RNA viruses within and across colonies. Among the mite-vectored viruses, the Deformed wing viruses (DWVs) are implicated in bee colony losses in Europe [3] and North America [4]. Understanding the genetic patterns behind resistance and tolerance to parasitic mites and the viruses they transmit is key for effective breeding programs aimed at reducing the risks and management costs of these threats.

We conducted three experiments to examine differences in gene expression patterns and virus infection levels in populations of honey bees with distinct genetic backgrounds and phenotypes - those tolerant of *Varroa* and resistant to honey bee viruses (R), and those susceptible to *Varroa* and/or viruses (S). Experiment 1 assessed natural virus infection loads and immune gene expression, using quantitative PCR (qPCR) to characterize differences between 15 R colonies, and 15 S colonies. Experiment 2 explored RNA sequencing data for gene-expression differences and pathogen levels distinguishing mite-infested bees of the R and S genetic backgrounds from sister bees that were verified to be mite-free. For Experiment 2, colonies were again classified as R or S prior to sampling, with the R colony having survived without acaricide treatment for more than 2 years as in Experiment 1, and the S colonies originating from a population known to be vulnerable to Varroa, and having been managed to control Varroa populations using conventional methods. RNA sequencing data revealed notable transcriptional differences between mite-infested and un-infested honey bees, and between Resistant and Sensitive phenotypes.

Reasoning that laboratory injections of bees with viruses would allow us to decouple gene expression patterns attributable to mite infestation from differences resulting from viral infection in field colonies, we conducted Experiment 3: injecting *Varroa-*free bee pupae in the laboratory with Deformed wing virus (DWV) or a phosphate buffer solution, and subsequently collecting RNA for sequencing. DWV injection evoked gene expression patterns that differed strongly from bees injected with only PBS. For Experiment 3 we defined R and S phenotypes by the changes in DWV copy number after DWV injection of pupae that were verified to be free of Varroa infestation. DWV levels were assessed by qPCR, and colonies that exhibited little or no change in DWV copy number after DWV injection were classified as R, while colonies that had elevated DWV levels after injection with DWV were classified as S. Importantly, this also provided an opportunity to uncouple transcriptional differences from genetic heritage as well as the variable field conditions and phenology of R and S phenotype classification in Experiment 2. Transcriptional profiles of virus-resistant R bees injected with DWV were markedly different from virus-sensitive S bees, while other gene expression contrasts emerged with buffer injection. The salient gene expression patterns that we observed in field and laboratory, under a variety of experimental conditions, demonstrate important differences between the two phenotypes in their response to *Varroa destructor* parasitism and Deformed wing virus infection. Equally important, our results allow differentiation of honey bee gene expression signals associated with viral infection in the presence and absence of mite parasites. These results have bearing on programs to understand host-parasite coevolution in a social insect and might be applied toward more sustainable strategies for reducing the impacts of parasitic mites on bees. They add support to predictions that bees surviving despite being subjected to unmanaged levels of *Varroa* mites do so because of their abilities to resist associated viruses [5, 6].

## Methods

### Experiment 1: natural responses in susceptible and resistant lineages

To assess the impacts of mite parasitism on gene expression and virus loads, 30 colonies were used. These colonies were on a migratory beekeeping path, spending fall, winter, and spring in Texas, and late spring through summer in Montana (where they were sampled). A total of 15 mite-resistant (R) and 15 mite-susceptible (S) cohorts were sampled. Susceptible bees came from colonies that were heavily infested with Varroa mites, e.g., phoretic mites were visible on adult bee and/or exhibited other symptoms such as parasitic mite syndrome; moreover, 13 of 15 S colonies also had visibly apparent deformed wing bees or other morphological disfigurements. R colonies had survived for more than 2 years without any chemical treatments or other interventions for mite control, and were headed by queens that were drawn from a population that had been managed without mite control interventions for more than 10 years at the time of the sampling. Both R and S colonies originated as packages with fewer than 1 mite per hundred bees, though the queens were, respectively, from a population exhibiting long-term survival without Varroa controls (R); and, from commercially available stock managed with typical Varroa controls (S). Sections of capped honey bee brood were cut from these 30 colonies. Worker honey bees were collected as they emerged from brood cells and these cells were simultaneously screened for the presence of *Varroa destructor* mites. Only bees from mite-free cells were utilized in Experiment 1, meaning these bees had never been directly parasitized by mites. Total RNA was extracted from individual bees using TRIzol® (ThermoFisher) following manufacturers’ protocol, generating ca. 50 μg total RNA per bee at 1 μg/μl. First-strand cDNA was generated from 1 μl of this RNA using random hexamer primers and Superscript II (ThermoFisher) following manufacturers’ protocol. Targets were screened using qPCR and appropriate primers for honey bee immune genes Nim2c, Hymenoptaecin and Eater, for viral pathogens Deformed wing virus, Black queen cell virus, and Kashmir bee virus, and for an endogenous control gene (*b-Actin*) using primers described in [7]. At the time of sampling only Deformed wing virus master variant ‘A’ was present in our population [8]. Data is presented as ΔCT, the relative gene expression of a target after normalization by the reference gene (*b-Actin*), with a higher number indicative of higher level of transcripts for that gene. ΔCT values were imported into the statistical software JMP (version 15 for MacOS), for individual *t-tests* and ANOVAs, as appropriate. In addition, clustering analyses were conducted to visualize overall divergence among samples and targets (Ward’s distance 2-way clustering, in JMP).

### Experiment 2: impacts of Varroa mites and natural DWV infection in susceptible and resistant bees

Sets of 30 parasitized and 30 mite-free bees were collected from two mite-susceptible honey bee colonies maintained at the USDA-ARS BRL, Maryland (S colonies), and from a single colony from a mite-resistant population (R colony). S colonies were typical commercial Italian stock and the R colony from a population that had survived without Varroa controls for more than 10 years at the time of sample collection. Both R and S bees showed roughly comparable levels of mite infestation, as revealed by observation of worker pupae for Varroa infestation as samples were collected for RNA extraction. Total RNA was extracted from individual bees, as above. A fraction of the extracted RNA was used to generate cDNA followed by qPCR reactions as in Experiment 1. Pools of RNA were generated using equimolar extracted RNA from the bees of each colony and approximately 8 μg total RNA was used directly to generate libraries for ILLUMINA paired-end 150 base-pair sequencing at the University of Maryland Institute for Genome Sciences. Bees from both mite-free cells and mite-infested cells were utilized in Experiment 2 and RNA sequencing data was reported, respectively, as R_control and S_control samples from mite-free cells, while bees from mite-infested cells were the source of material for RNA sequencing data for R_mite and S_mite samples. R and S samples of Experiment 2 were drawn from different populations with pre-defined phenotypes of Varroa and virus resistant (R) and Varroa and virus sensitive (S).

### Experiment 3: impacts of deformed wing virus on resistant and susceptible bees

Intact honey bee brood frames were collected from each of 10 colonies maintained in apiaries near Navasota, Texas.

30 white-eyed pupae were removed from mite-free brood cells in brood comb. We individually screened and evaluated all pupae, and the capped brood cells containing them, for evidence of mite infestation, as in Experiments 1 and 2. Consequently, while we report no colony-level data regarding mite infestation levels or mite loads, we have reliable information regarding the Varroa parasitism status of individual bees in all three experiments reported here, a more accurate metric than imputed infestation probabilities from colony level mite loads. Bees were injected with 1 μl of PBS alone, or PBS containing ca. 10^7^ DWV viral copies. DWV suspensions for injection were prepared by extracting hemolymph from adult worker honey bees from the USDA-ARS Bee Research Laboratory apiaries (Beltsville, MD) showing the pathology of DWV infection, deformed wings. After virus injection, pupae were allowed to develop for 48 h on folded Whatman paper in petri dishes incubating at 34 °C with controlled relative humidity. Total RNA was extracted from 15 bees either injected with PBS or DWV. Quantitative PCR was performed on aliquots of RNA from individual bees using primers for Hymenoptaecin, Eater and DWV, as in Experiment 1. RNA from bees of two colonies showing higher mean DWV levels after injection (Susceptible, S), and RNA from bees of two colonies with stable mean DWV levels after injection (Resistant, R) were pooled for RNA sequencing. Pools of RNA were generated using equimolar extracted RNA from the bees of each colony, and approximately 8 μg total RNA was used directly to generate libraries for ILLUMINA sequencing, as above. Experiment 3 samples assigned experimentally-defined phenotypes of virus resistant (R) as well as samples designated as virus sensitive (S) were from different source populations that did not share recent genetic heritage, insuring that the results of Experiment 3 are not merely the reflection of genetic differences in gene expression of R and S samples.

### Statistical analyses of RNA sequences

Raw sequence reads in FastQ format were trimmed using Trim Galore. Remaining adapter sequences were removed and sequences with quality scores < 25, as well as reads < 35 bp, were also removed. Trimmed FastQ files were aligned to the Honey Bee (*Apis mellifera*) Genome Amel HAv3.1 using HiSAT2 [9] or STAR [10]. Features (genes, transcripts and exons) were counted using featureCounts [11] and StringTie [12] using the GCF_003254395.2_Amel_HAv3.1 gene annotations [13]. Reads not aligning to the honey bee genome were subsequently aligned to the HolobeeBar microbial database (https://data.nal.usda.gov/dataset/holobee-database-v20161) using Bowtie 2 [14]. Basic pairwise differential expression analysis was performed using EdgeR [15] and DESeq2 [16].

Differential gene expression patterns were derived from RNA sequencing data using read alignments and read counts from the DESeq2 package. Genes showing up-regulation or down-regulation were compiled for all pairwise comparisons of genetic background (R v. S) and biological condition, mite-infested or mite-free and virus-injected or buffer-injected. Results were filtered for all evaluated contrasts by excluding any gene that had a False Discovery Rate (FDR) greater than or equal to 0.05. Increased gene expression levels (UP) or decreased gene expression levels (DOWN) compared to an expression standard (*b-Actin*) are limited to those genes with a FDR < 0.05 and that have a log2 Fold Change (logFC) of > + 1.5, or < − 1.5 for UP-regulated expression and DOWN-regulated expression, respectively.

Unfortunately, many honey bee genes remain poorly characterized, and while gene expression responses to the environmental and experimental conditions examined here are inevitably complex, these factors impede interpretation of our differential gene expression results. Thus, to enrich insights gleaned from our differential gene expression results, we employed HymenopteraMine [17], a sophisticated genomic data analysis environment. HymenopteraMine facilitated exploitation of gene annotation information, simplified comparison of genes sets differentially expressed in the many combinations of genetic lineage and experimental conditions and enabled gene ontology enrichment (GO enrichment) analyses.

We engaged gene ontology (GO) annotations and the GO biological process, molecular function and cellular compartment enrichment widgets (GO BP, GO MF and GO CC) available at HymenopteraMine to analyze the genes UP and DOWN in S_mite v. R_mite, genes UP or DOWN in S_virus v. R_virus, and genes differentially expressed in other sample comparisons. When GO enrichment results were available, they were used to provide insights into the gene expression patterns that distinguished resistant and susceptible bees under the biological conditions we evaluated using RNA sequencing data - natural mite infestation and latent viral infection, and laboratory injection of DWV virus or a phosphate buffer solution. We displayed results using available tools (e.g., ReviGO for visualizing Gene Ontology patterns, http://revigo.irb.hr/) and custom developed scripts.

## Results

### Experiment 1: evaluation of susceptible (S) and resistant (R) colonies for virus loads and immune gene expression levels

**Fig. 1:**
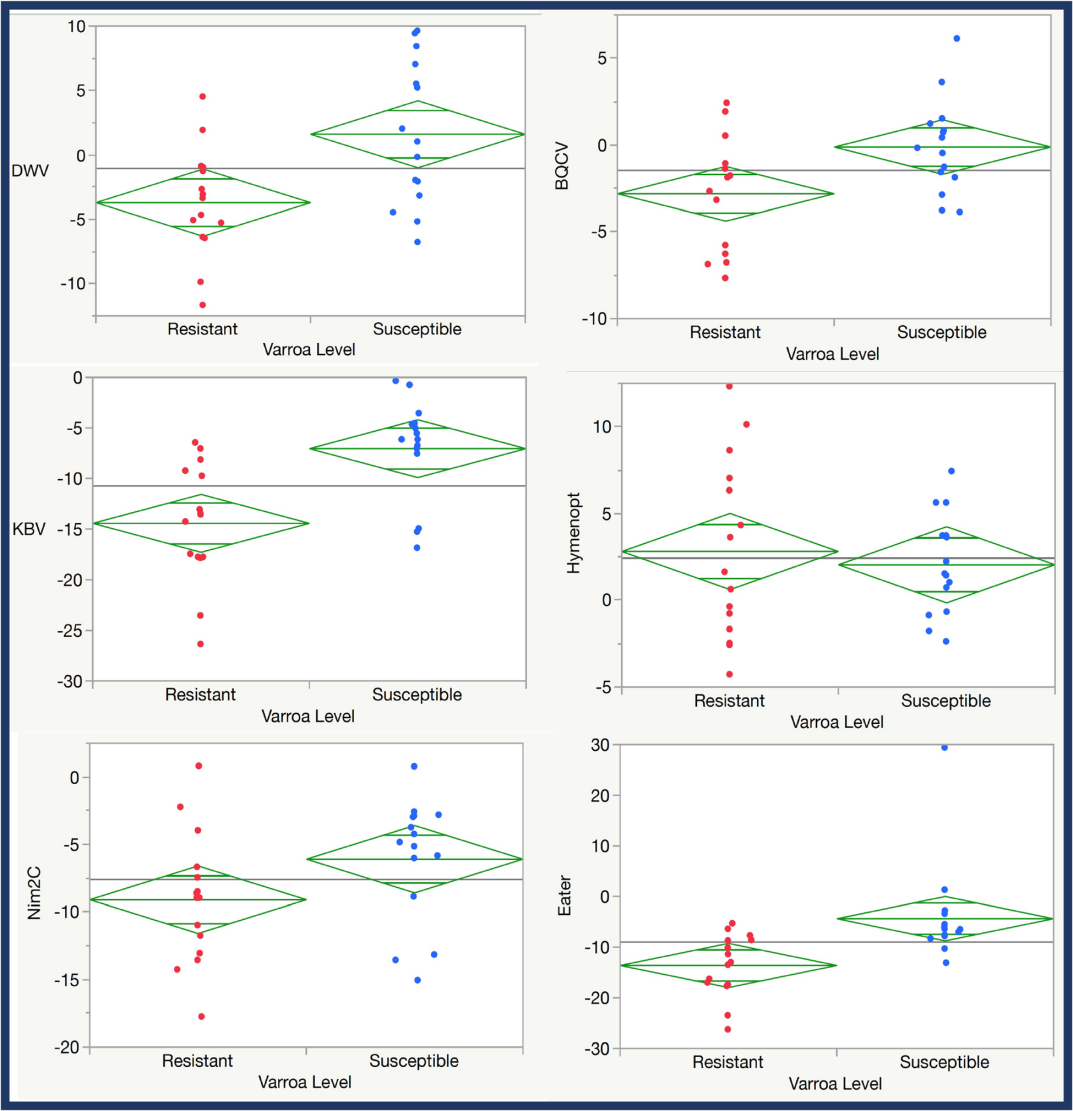
Natural virus loads of Deformed wing virus (DWV), Black queen cell virus (BQCV), Kashmir bee virus (KBV), and expression levels of three immune genes: the antimicrobial gene, Hymenoptaecin, and the cellular immunity genes, Eater, and Nim2C. Diamond plots show means as the center line and a midline for one standard deviation, while the points reflect 95% confidence intervals for the mean for each target

**Fig. 2:**
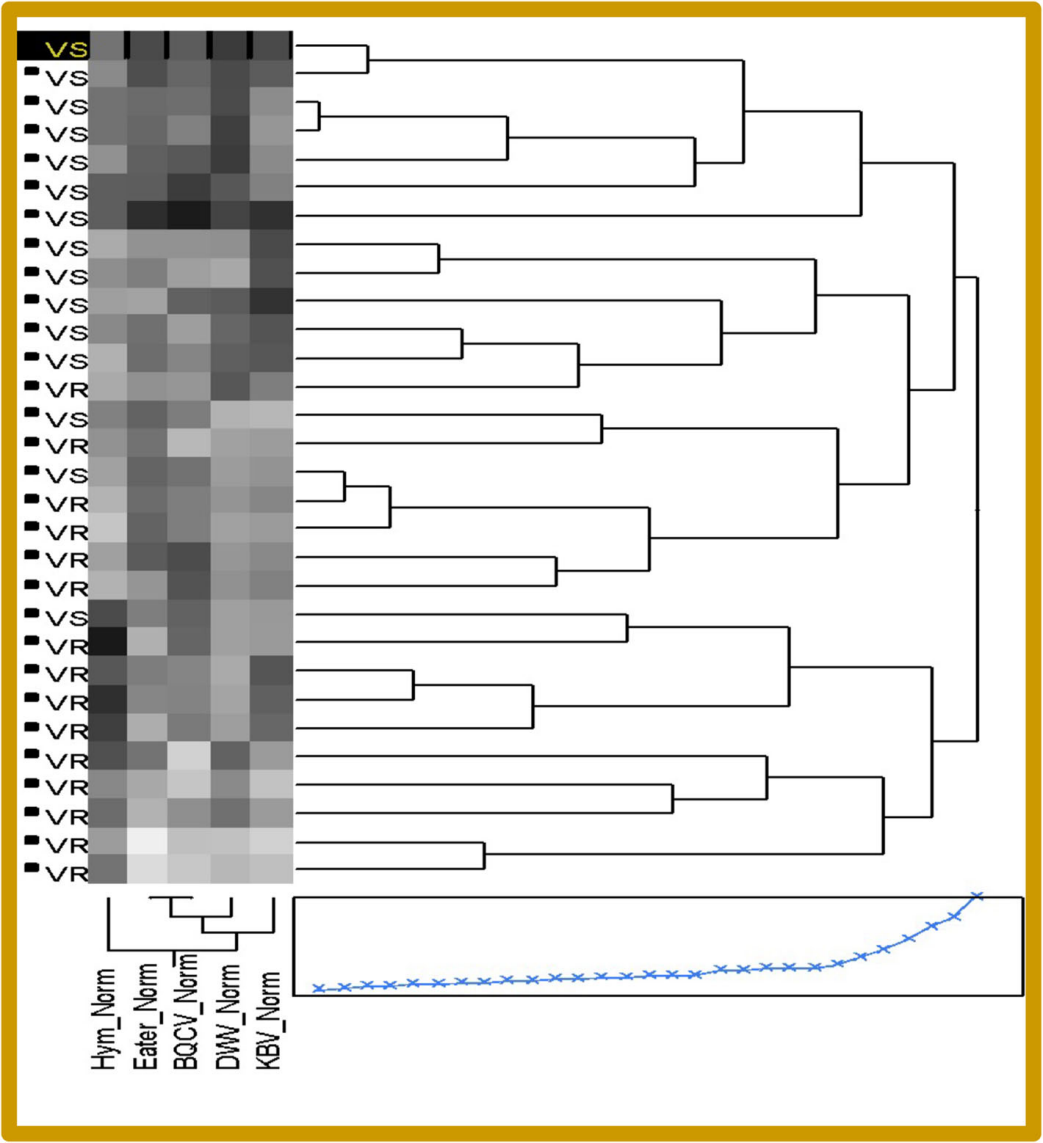
Clustering Diagram of Susceptible (VS) and Resistant (VR) samples from Experiment One, showing higher virus loads in VS bees than in VR bees and higher levels of Eater expression in VS bees, contrasted with slightly higher levels of Hymenoptaecin expression in VR bees

Colonies from both lineages lacked pathologies typical of highly infected honey bees. To determine whether these lineages differed in gene expression and viral loads, colonies were screened using quantitative-PCR (qPCR). Comparing qPCR data of bees from R colonies to that of bees from S colonies, we observed that R bees exhibited much lower natural levels of Deformed wing virus (DWV), as expected (Fig. 1). These bees also showed decreased levels of Black queen cell virus (BQCV), a virus not transmitted by mites, and Kashmir bee virus (KBV), than did S bees. S bees also expressed much higher levels of the cellular immunity genes Eater and Nim2C than did R bees. Strikingly, resistant bees showed higher levels of the gene encoding the antimicrobial peptide Hymenoptaecin and this was a major driving force in separating the two sample classes by cluster analysis (Fig. 2 and Fig. S1).

### Experiment 2: gene expression in R and S genetic backgrounds, with and without Varroa mites

**Fig. 3:**
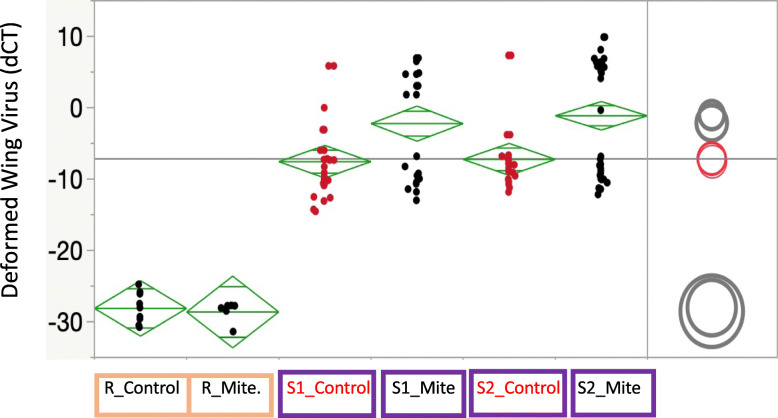
Quantitative PCR estimates of Deformed wing virus (d-CT) for the R_control, R_mite, S_control, and S_mite samples, showing mite-infested Susceptible honey bees with elevated DWV titers compared to Susceptible bees that were mite-free. Susceptible bees (two sources) had markedly higher levels of DWV than the Resistant bees, regardless of Varroa parasitism status; moreover, DWV loads of Resistant bees were not higher when mites were present. Diamond plots show means as the center line and a midline for one standard deviation, while the points reflect 95% confidence intervals for the mean for each target. Confidence circles to right show pairwise t-test differencs at *p* < 0.05. Orange samples reflect resistant bees, purple samples reflect susceptible bees

Honey bees from the R population did not show an increase in viral levels with Varroa presence (Fig. 3), in contrast to the two S samples. Both mite-free and parasitized bees had significantly lower mean viral levels than the mite-free and mite-parasitized susceptible bees (*p* < 0.0001 for all pairwise comparisons). In the susceptible colonies, mite-parasitized bees had significantly higher viral loads (*p* < 0.0001 for Susc364 and *p* = 0.0021 for Susc309). We used RNA sequencing to compare gene expression levels between R and S bees collected from field colonies under natural conditions, comparing transcriptional profiles of R bees with and without Varroa mites to S bees with and without mites. Gene-expression signals were analyzed for statistically meaningful differential expression using DESeq2. Increased gene expression levels (UP) or decreased gene expression levels (DOWN) compared to an expression standard (*b-Actin*) are limited to those genes with a FDR < 0.05 and that have a log2 Fold Change (logFC) of > + 1.5, or < − 1.5 for UP-regulated expression and DOWN-regulated expression, respectively. We sequenced RNA of bees from R and S colonies, both from bees with mites present (R_mites; S_mites), and from bees without mite infestation (R_control; S_control). Strikingly, 89% of the variance in differential gene expression among bees from S and R colonies, under the two biological conditions of Experiment 2 (with and without mites), was explained by Principal Component 1 (74%) and Principal Component 2 (15%) in a Principal Component Analysis (Fig. S2). Transcriptome-wide analyses tend to cluster both mite and control samples from resistant bees, while showing a sharp disparity in gene expression between the two conditions for the susceptible colonies, highlighting the impacts of mite presence on those lines (Fig. S1).

**Fig. 4:**
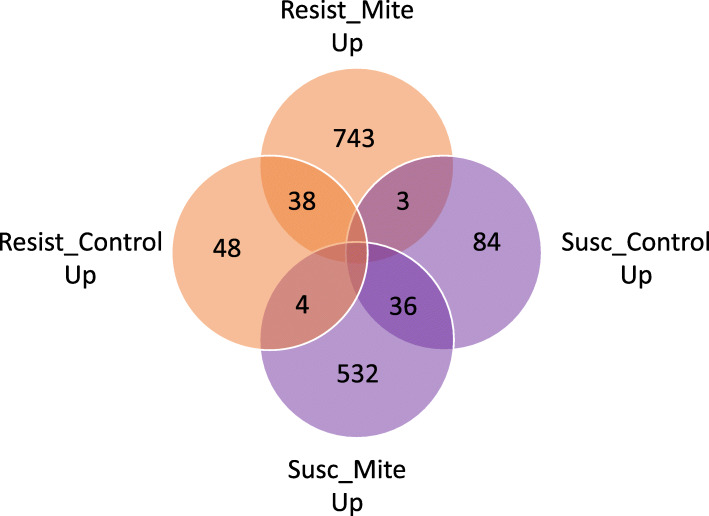
Venn diagram showing differentially expressed genes in Resistant and Susceptible stock with and without the presence of parasitic mites

**Fig. 5:**
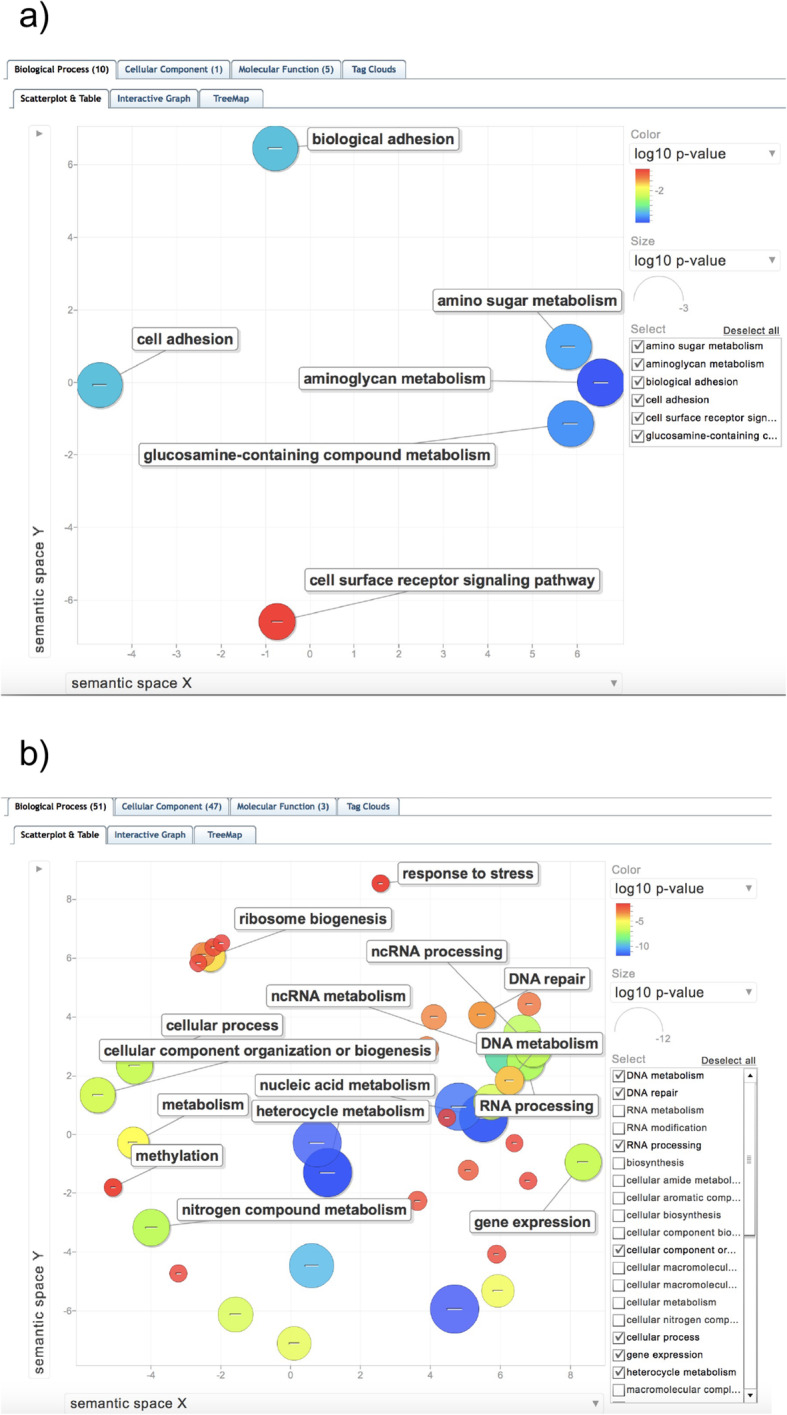
Scatterplot of enriched Biological Process Gene Ontology terms (GO BPs). a) Up-regulated in S bees with mites compared to R bees with mites (UP in S_mite v. R_mite), b) Down-regulated in S bees with mites compared to R bees with mites (DOWN in S_mite v. R_mite). GO enrichments and *P*-values were calculated using HymenopteraMine GO enrichment widgets after using HymenopteraMine database cross-references to convert gene identifiers to OGSv3.2, with the OGSv3.2 gene set as the background population and a Benjamini-Hochberg multiple testing correction. The GO enrichment test dataset is comprised of genes with FDR < 0.05 and with expression level differences showing a log10 fold change greater than 1.5 (logFC> 1.5). Scatterplot generated by ReviGo [18]

In total, 847 honey bee genes were expressed at higher levels in S bees with mites than in R bees with mites (UP in S_mite v. R_mite), including miR-3726 and miR3729 with 9.51 and 13.87 log fold elevation in S_mite over R_mite, respectively (Fig. 4, Supplemental Table 1). Several immune effectors were among those overexpressed genes in S_mite v. R_mite samples, including the effectors Apidaecin, Defensin-1, and Hymenoptaecin, and two peptidoglycan recognition proteins (GB47805 and GB47804). Differentially expressed genes up-regulated in S bees exposed to mites compared to R bees exposed to mites have GO enrichment results for the Gene Ontology biological processes *cell adhesion*, *cell surface receptor signaling* and *biological adhesion*, as well as *aminoglycan metabolism* and *glucosamine compound metabolism* (Fig. 5). Cell adhesion molecules and other cellular membrane components are widely implicated as receptors for viral entry into host cells, and subsequent cell receptor signaling aids viral internalization and hijacking of cellular machinery for virus replication [19, 20]. Some aminoglycan derivatives can serve as receptors for virus entry as well as cell and membrane adhesion molecules. Chitin, a principal component of the exoskeleton, is a structural aminoglycan, and Varroa mites must penetrate chitin with their mouthparts to feed on honey bees, and bees must repair that damage to avoid desiccation, infection by microbial species and death. The increased expression of genes involved in these biological processes in mite-susceptible and virus-sensitive S bees have obvious implications in promoting vulnerability to virus infection and compensating for damage inflicted by mite parasitism.

The 920 genes UP-regulated in R_mite v. S_mite have GO BP enrichments for nucleic acid metabolism, RNA processing, non-coding RNA metabolism, gene expression, DNA metabolism, DNA repair and cellular response to DNA damage stimulus, mitochondrial gene expression, peptide metabolism and peptide biosynthesis, translation, methylation and cellular response to stress (Supplemental Table 2). 24 genes related to cellular response to DNA damage and DNA repair that are elevated in R_mite v. S_mite are apparent homologs or orthologs of dipteran and mammalian genes involved in all distinct DNA repair mechanisms: mismatch base repair, nucleotide and base excision repair, interstrand crosslink repair, non-homologous end-joining and homologous recombination; as well as genes affecting cell cycle arrest and DNA damage checkpoints. 10 of these 24 genes are also UP in R_mite over R_control expression levels. This suggests that DWV and Varroa radically disrupt normal gene expression activity and may inflict substantial damage to host DNA. At least one positive-strand RNA virus in humans - Hepatitis C virus - causes significant DNA lesions in hepatocytes, and there are many more examples of viruses generally with oncogenic/mutagenic activity. Fundamental metabolic changes differentiating R and S bees affected by Varroa and DWV, especially gene expression program alterations, nucleic acid synthesis, and DNA repair processes, that could compensate for the various pathogenic disruptions of DWV and Varroa, may be key to the enhanced viral and Varroa resistance of R bees. The complete list of GO BP enrichments from genes UP in R_mite v. S_mite is given in Supplemental Table 2, Sheets 1 and 2.

Genes up-regulated in S_control v. R_control, where mites are absent, also reflect many GO BP enrichments. Some of the more interesting indicate that energy metabolism, ribonucleoside and nucleotide synthesis, and translation of mRNA to protein are all elevated in S over R bees without mites. The GO Biological Process enrichments are listed in Supplemental Table 2, Sheet 3. For 107 genes UP in R_control v. R_mite, GO enrichments include regulation of transcription, regulation of macromolecule biosynthetic process, regulation of gene expression, cellular adhesion processes, regulation of RNA metabolic processes, and transcription factor activity among others, and are described in Supplemental Table 2, Sheet 4.

Surprisingly, there are very few genes differentially expressed when comparing S_control to S_mite, and in fact there are none UP in S_control over S_mite; so, a general down-regulation of gene expression by Varroa finds no support in our evidence. There are only 16 OGSv3.2 genes DOWN in S_control v. S_mite, yielding no GO enrichment results for that comparison. The most notable genes down-regulated in S_control v. S_mite - that is, genes with elevated expression in S when Varroa are present are: Cytochrome P450 6A1, Fibroin heavy chain, IL-1 receptor, lactate dehydrogenase, and one of five genes identified as a homolog or paralog of protein lethal (2) essential for life - GB45910 (724367), plus Hymenoptaecin, and bone morphogenetic protein 2-B.

### Experiment 3: differential gene expression of resistant and sensitive bees after injection with DWV or a saline control

**Fig. 6:**
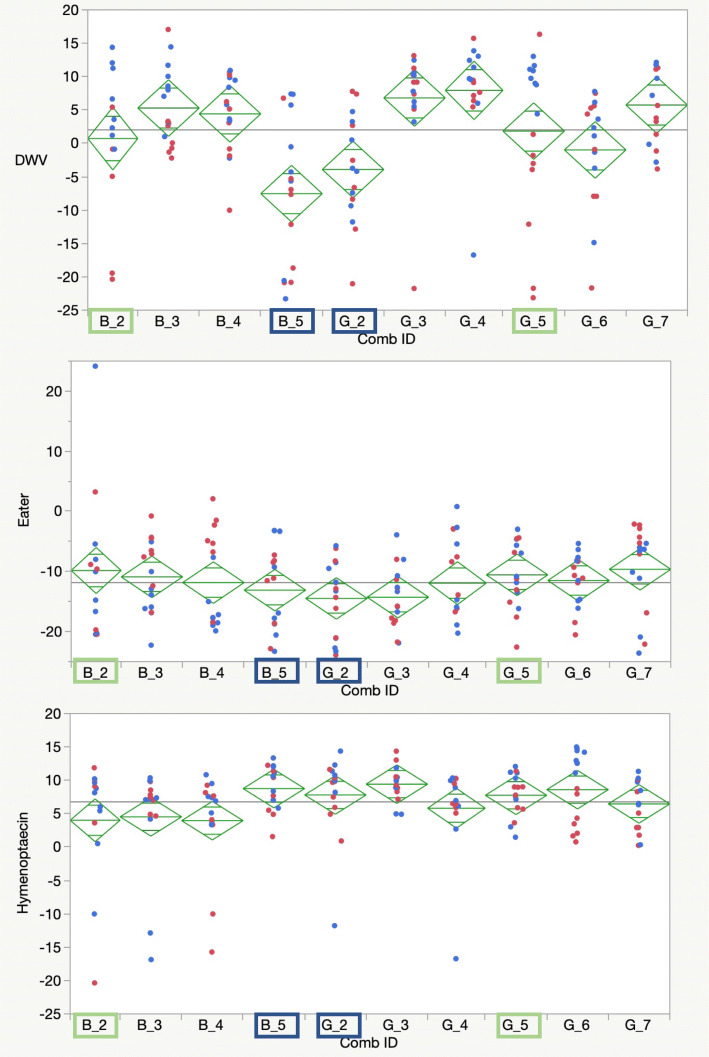
qPCR relative levels of Deformed wing virus and two immune-related genes, Eater and Hymenoptaecin. Blue dots reflect bees injected with DWV, red dots reflect bees given a PBS control injection. Samples highlighted in blue were chosen for sequencing as resistant bees due to limited viral growth, samples highlighlighted in green were sequenced as susceptible bees. Diamond plots show means as the center line and a midline for one standard deviation, while the points reflect 95% confidence intervals for the mean for each target

Viral loads and immune gene expression differed significantly across colonies for bees injected with live DWV and those injected with PBS (Fig. 6 and Fig. S4). Pupae from some colonies showed high viral loads with either condition (e.g., sources B4, G3, G4, and G7, statistics in Fig. S4, B), suggesting high existing levels of viral infections that were arguably amplified regardless of the addition of new viral copies. Others (B2, B3, B5, G5) showed significant increases in viral loads when bees were injected with live virus. Two sources (B5 and G2) held relatively low average viral titers with both control and live virus injections.

Samples of pooled half-sisters from colonies that exhibited little or no increase in mean DWV titers after DWV injection (virus resistant (R)), were compared to samples of pooled half-sisters that demonstrated increases in mean viral loads after DWV injection, (virus sensitive (S)). Again, strong contrasts were revealed when examining comparative responses to virus injection in R and S genetic backgrounds, and other disparate gene expression differences emerged when comparing the response to PBS injection. Both the identity of genes exhibiting expression level changes, and the direction of expression level change (elevated or reduced expression - UP or DOWN) differed among Varroa-free bees drawn from Resistant and Sensitive colonies, as shown by Principal component analyses (Fig. S2).

### Genes expressed more in R_virus than in S_virus

**Fig. 7:**
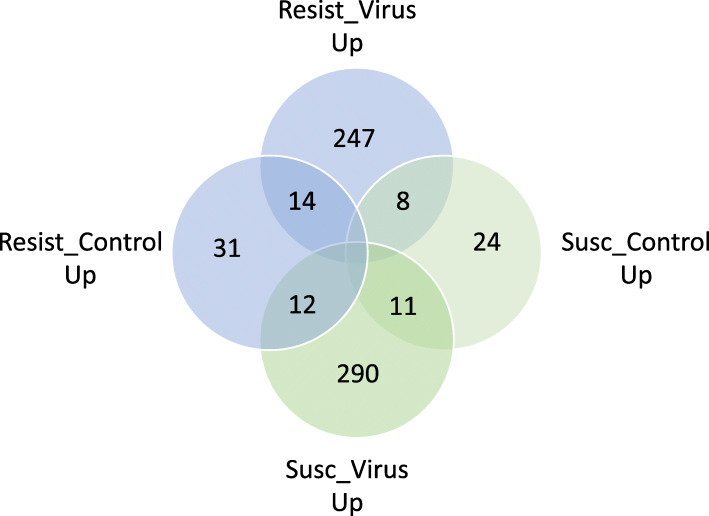
Venn diagram showing differentially expressed genes in Resistant and Susceptible stock with and without injection with Deformed wing virus

269 genes were UP in R_virus over S_virus (Fig. 7), leading to GO BP enrichments for defense response, oxidation-reduction processes, immune response, innate immune response and immune system process (Fig. S3; Supplemental Table 2, Sheet 6). However, 75 of these genes had no GO BP annotation. The genes producing the GO BP enrichment for immune response, immune system process and defense response included Defensin-1 (GB41428), peptidoglycan recognition protein 1 (GB47804), peptidoglycan recognition protein-2 (GB47805), leucine-rich repeat-containing protein 26 (GB44192), Hymenoptaecin (GB51233), and GB54506, an uncharacterized protein having scavenger receptor activity, binding acetylated and oxidized LDLs, bacteria, apoptotic cells, and advanced glycation end products, and delivering those ligands into the cell. The genes expressed at higher levels in R bees injected with virus than S bees injected with virus also yield GO molecular function enrichment for endopeptidases and other functional enzymes (Supplemental Table 2, Sheet 7).

Vitellogenin (GB49544); Pla2 or Phospholipase a-2 (GB48228) a venom component; PGRP-S2 (GB47805) encoding a peptidoglycan recognition protein; SP34 (GB48510), a venom serine protease; and Est-6 or carboxylic ester hydrolase (GB53756) were all expressed at higher levels in R_virus than S_virus. Among immune genes, the immune effector Apidaecin is also among the top 50 more elevated in R_virus v. S_virus with log2 Fold Change greater than 3 and Defensin-1 (GB41428) is also among the more highly expressed in R bees compared to S bees injected with virus, both with log FC > 2.9. PPO or Prophenoloxidase (GB43738) is also among the top 100 most differentially expressed and elevated in R_virus over S_virus. Lastly, Argonaute-2 (GB50995) is UP in R_virus v. S_virus, as is GB41545 (LOC409187), or MD-2-related lipid recognition protein involved in cholesterol transport, and both have been implicated in anti-viral defenses in honey bees [21]. Additional genes of interest are discussed in Supplemental Text 1.

### Genes strongly down-regulated in R_virus v. S_virus injected bees

Apidermin-3 like protein has recently been implicated as an outlier protein suppressed by DWV but induced by Varroa parasitism in a proteome study [22]. In that report Apidermin appears to be down-regulated by DWV infection, but up-regulated by Varroa and DWV + Varroa. We find that DWV injection suppresses Apidermin-3 like protein GB53110 (409716) in R bees that are mite-free and in S bees that are mite-free. That is, GB53110 is DOWN in R_virus v. R_PBS and DOWN in S_virus v. S_Control. But Apidermin-3 is also DOWN in R_virus v. S_virus - in substantial agreement with [22]. It appears that Apidermin 3 (GB53110) is up-regulated in S bees generally despite mite infestation and/or DWV infection. Alternatively, Varroa and Varroa+DWV may up-regulate Apidermin-3 only in S bees.

The MAGE-like protein (LOC102654246) encoding gene, GB42910, is another example of one of the most down-regulated in the R_virus v. S_virus contrast, and is often involved in stress response in other species. Similarly, the gene encoding the *Ctenidin 1-like* protein (LOC102656669) is another gene most DOWN regulated in R_virus v. S_virus, and codes for a glycine-rich protein orthologous to an antimicrobial peptide first described in spiders and scorpions. Interestingly, *kakusei*, a gene transcribed into a non-coding RNA, known to be an Immediate Early Gene (IEG) and previously characterized as involved in associative learning and memory and other neural activity, is also a gene DOWN in R_virus v. S_virus. IEGs are also recognized as one of the key mediators of links between events at the cellular membrane and the nucleus, presumably related to neural activity.

The Hexamerin 110 encoding gene GB44996 is strongly down-regulated in R_virus v. S_virus. Hex110 is an amino acid storage protein used to fuel the drastic changes that occur during metamorphosis, and Hexamerin 110 may have a role in regulating the expression of the ribosomal RNA gene cluster in the nucleolus. Hex70a, Hex70b and Hex70c are also DOWN in R bees relative to S bees injected with virus, though not so dramatically as Hex110. Other notable genes DOWN in the R_virus v. S_virus contrast include Kr (GB41483) or Krueppel, and Usp (GB42692) or Ultraspiracle. Krueppel is a chromatin-associated gap class segmentation protein that is involved in negative regulation of transcription and developmental processes, including hemocyte proliferation, trunk segmentation and neurogenesis. Ultraspiracle is a nuclear steroid hormone receptor, binding ecdysone and ecdysone response elements, and is probably involved in honey bee metamorphosis. GB50662 (Vhdl), a larval-specific very high-density lipoprotein, is also Down in R compared to S bees injected with virus.

## Conclusions

Honey bees battle their parasites with both individual and group defenses. Honey bees that survive long-term exposure to *Varroa* mites without the help of human interventions are exceedingly rare. These surviving lineages could resist mites themselves or the devastating viruses that mites vector within the colony [5, 6]. The actual genetic mechanisms behind this resistance are elusive, and understand them is a critical step in identifying and maintaining desirable honey bee populations. The three described experiments focused on signals for both traits, namely genetic responses by bees that reduce the impacts of mites and mite-vectored viruses.

Experiment 1 showed that natural viral loads in a genetically distinct population of honey bees [23] exhibiting a Varroa tolerant phenotype were markedly lower than natural viral loads in another population of bees susceptible to Varroa. This trend of lower virus levels, including a virus not vectored by *Varroa* mites, suggests a general resistance to viruses. Expression levels of the cellular immunity genes *Eater* and *Nim2C* were elevated in susceptible bees compared to resistant bees. These results led us to more closely examine differences between these two populations, and their respective responses to *Varroa destructor* parasitism and virus infection.

In Experiment 2, we generated RNA sequencing data from R and S bees kept under natural conditions for commercial beekeeping and queen-rearing purposes. These data confirmed the results of Experiment One - that R bees harbor lower covert viral loads, including DWV, than do S bees. Our results also indicate that mite infestation appears not to elevate viral titers in R bees, while mite infestation is associated with significantly higher virus levels in S bees. Previous work has shown that Varroa parasitism consistently results in markedly elevated DWV levels and manifestations of DWV pathology (*See,* e.g.*,* [24]). This suggests that the Varroa tolerance of R bees may be explained at least in part by R bees suppressing the viral load enhancement that usually follows parasitism by Varroa. Alternatively, the virus resistance of R bees may be related to their ability to cope with Varroa infestation without suffering pathological effects, including aberrant immune system function, and increased mortality usually associated with mite-induced viral load enhancement. Finally, the Varroa tolerance and lower viral loads in R bees compared to S bees may be driven by a combination of enhanced resistance of R bees to both Varroa and viruses, with distinct expression differences conferring resistance both to pathogens and a pathogen-vectoring parasite.

Especially intriguing in Experiment 2 are the GO enrichments for RNA processing, including transcription and transcriptional regulation, splicing, nuclear and mitochondrial gene expression, translation and peptide biosynthesis, plus DNA damage repair. The fundamental metabolic changes that distinguish the response of R and S bees to DWV and Varroa mites may better enable R bees to cope with the insults of Varroa and viruses and their deleterious effects on transcription, translation, gene expression programs and nucleic acid processing. The differential responses to Varroa and DWV also encompass extensive activation of DNA damage repair pathways by R bees. Overall, R bees have higher levels of expression for genes implicated in DNA and RNA processing, especially splicing of RNA, gene expression and nucleobase and ribonucleoprotein metabolic processes. R_PBS bees also express Argonaute-2 GB50955 at higher levels than S_PBS bees (DOWN in S_PBS v. R_PBS). This protein is also up-regulated in R_virus v. S_virus samples. Argonaute-2 is a key component of the RNA interference response, a key antiviral pathway [25], and this pathway has been shown to be responsive to viral infections recently by Rutter et al. [21].

Experiment 3 was designed to decouple the direct effects of mite parasitism from the effects of transmitted viruses. Specifically, we examined differentially expressed genes from mite-free bees of both R and S phenotypes and from common locales, by directly injecting DWV or PBS into bees of both genetic backgrounds. When comparing the changes in gene expression elicited by virus injection, the results clearly differentiate R and S bees. UP regulation of immune response genes in R_virus v. S_virus suggests that elevated expression levels for genes involved in defense response and immune response may be an effective response to viral infection, conferring some degree of protection against DWV, at least in honey bees free of Varroa infestation. Elevated expression of immune response genes differentiate R bees from S bees in response to direct DWV injection and may be an important advantage contributing to the relative resistance of R bees to DWV. However, as Experiment 2 results reveal, when Varroa mites are present, turning up expression of immune system genes alone may not be sufficient for coping with Varroa and natural virus infection. Our GO enrichment results show that S bees infested with mites, and carrying elevated DWV loads compared to R bees with mites, do upregulate some immune genes. But if up-regulation of immune genes was sufficient to confer a virus and mite resistant phenotype, then we might expect to see immune gene expression generally elevated in R_mite v. S_mite, while the GO enrichment data show the opposite. A more complex response to mites + virus, selectively modulating expression of many genes but enhancing expression of only specific immune genes, may contribute to increased mite-tolerance and virus resistance in R bees.

Many interesting GO terms emerged from genes with elevated expression after DWV injection, as well as from genes up-regulated in association with natural DWV infection, contrary to the complete absence of GO enrichment among genes elevated by pathogen infection in Doublet, et al. [26]. In an especially striking example, our experiments show that genes involved with immune function were expressed at higher levels in samples with higher DWV loads - a result at odds with the meta-analysis of Doublet, et al. [26], where immune genes, metabolic genes and regulatory genes were all suppressed by pathogen infection. Most notably, we find immune genes and defense response genes were highly over-represented among genes UP in R bees after virus injection, and were also UP in S bees with mites. Enhanced expression of immune defense genes elicited by higher DWV load is one explanation for our results: S bees with mites harbored higher levels of natural DWV infection than R bees with mites or R bees without mites. Equally important, R bees expressed immune defense genes at higher levels but developed lower DWV loads after DWV injection. These results offer intriguing correlations with the differential response of R and S bees to DWV injection, as well as their response to natural DWV infection in conjunction with Varroa infestation. Elevated expression of select immune genes may represent an effective anti-viral response to DWV infection, albeit one modulated by Varroa or of reduced impact when mites are present.

### Integrating differential gene expression results of bees with natural DWV infection but variable Varroa infestation from expression differences after DWV injection of mite-free bees

The disparate patterns of elevated gene expression between R and S bees naturally infected with DWV and infested by Varroa mites, compared to differential expression of genes in R and S bees infected in the laboratory with DWV, potentially provide clues to the mechanisms of resistance to virus and tolerance of mites. The totality of the evidence suggests that R bees devote significant energy to buffering against cellular and metabolic stress that emanate from concomitant virus and parasite insults, and exhibit differential gene expression patterns that provide metabolic, oxidative and developmental stress protections - not simply elevated immune gene expression profiles. While elevated immune system gene expression may be necessary for successfully resisting viral infections, increased expression of immune genes alone may be insufficient to provide protection, especially in bees with both DWV infection and *Varroa destructor* infestation.

### Genes also differentially expressed in previous studies

We found concordance in differentially expressed genes between our study and four RNASeq analyses conducted on honey bees and their disease responses, as detailed in Supplemental Table 3. On the other hand, we find important differences between our results and prior reports of particular genes being best correlated with DWV infection or antiviral response. In fact, in several instances genes previously identified as key anti-virus responses were not differentially expressed in our study [21, 22, 26–32].

The salient gene expression patterns that distinguish R and S bees in the field and laboratory demonstrate important differences between the two genetic backgrounds in their response to *Varroa destructor* parasitism and DWV infection. The gene expression patterns associated with mite infestation and DWV infection provide additional insights into important transcriptomic changes elicited either by direct virus injection and infection, or by Varroa mite parasitism and natural viral infection. Equally important, our results show different honey bee gene expression signals are elicited by viral infection depending upon the presence or absence of mite parasites. We recovered a robust signal of differential gene expression when comparing bees from two genetically distinct populations exposed to DWV and Varroa mites, despite sampling polyandrous bees with the intra-colony genetic diversity typical of natural hives. Our data may also reveal responses to viral infection and mite parasitism that confer selective advantages in bees that exhibit mite-tolerant and virus-resistant phenotypes, and may suggest mite tolerance or virus-resistance mechanisms. This work should allow us to begin identification of gene expression patterns associated with mite tolerance and viral resistance in populations or lineages that exhibit those traits, and begin to define virus and mite resistance mechanisms at the genomic and transcriptomic level – all of which merit additional investigation.

## Supplementary Information

**Additional file 1 **: **Fig. S1**. Heatmap of the sample-to-sample distance in R_mite v. R_control and S_mite v. S_control samples compiled using the R package DESeq2 with a customized map function, (http://bioconductor.org/packages/release/bioc/vignettes/DESeq2/inst/doc/DESeq2.html#heatmap-of-the-sample-to-sample-distances). **Fig. S2**. A) Principal Component Analysis of Gene Expression Data produced by DESeq 2 for R_mite, R_control, S_mite, and S_control. Note that PC1 captures 74% of the total variance between the samples, and shows the extreme divergence of R_mite from S_mite and S_control. PC2 provides differentiation of R_control from R_mite, revealing much of the gene expression differences attributable to mite infestation of the R genetic background. B) Principal Component Analysis of Differential Gene Expression data, contrasting R bees injected with virus, R_Virus, S bees injected with virus, S_Virus, and R & S bees injected only with a phosphate buffer solution, R_PBS and S_PBS; R_Virus and S_Virus are divergent in RNA sequencing data that comprise principal component 1; Principal component 2 captures the different responses to virus versus control injection in the R samples, and differences between the two S samples in response to buffer injection. **Fig. S3**. a) Scatterplot in semantic space of GO BP enrichment for UP in R_virus v. S_virus, FDR < 0.05 and logFC> 1.5. b) Scatterplot in semantic space of GO MF enrichment from HymenopteraMine set operation of Asymmetric Difference of UP in R_virus v. S_virus *MINUS* UP in R_virus v. R_PBS, genes with FDR < 0.05 and logFC > 1.5. **Fig. S4**. A) Mean Deformed wing virus levels separated by colony and by injection of viruses versus control (PBS). Diamond plot points indicate 95% confidence intervals B) Individual means and standard error estimates for each set of eight bees by colony and virus exposure.

**Additional file 2 **: **Supplemental Table 1**: Fold-change differences for genes found to be significant under a false-discovery cut-off of 0.5, shading reflects strength of fold change increasing from yellow to red.

**Additional file 3 **: **Supplemental Table 2**: Raw Gene Ontology results for diverse comparisons across the two experiments using RNA sequencing.

**Additional file 4 **: **Supplemental Table 3**. Common genes identified in multiple studies that measured impacts of disease on honey bee gene expression.

**Additional file 5 **: **Supplemental Text 1**. Additional textual highlights of our results regarding individual genes found associated with honey bee mite and virus interactions in prior work.

## Data Availability

All described sequences are stored in the NCBI Sequence Reads Archive (https://www.ncbi.nlm.nih.gov/nuccore/1444462244)..

## References

[1] Boncristiani H, Ellis JD, Bustamante T, Graham J, Jack C, Kimmel CB, Mortensen A, Schmehl DR (2021). World honey bee health: the global distribution of Western honey bee (Apis mellifera L.) pests and pathogens. Bee World.

[2] Ramsey SD, Ochoa R, Bauchan G, Gulbronson C, Mowery JD, Cohen A, Lim D, Joklik J, Cicero JM, Ellis JD, Hawthorne D, vanEngelsdorp D (2019). Varroa destructor feeds primarily on honey bee fat body tissue and not hemolymph. Proc Natl Acad Sci U S A.

[3] Dainat B, Evans JD, Chen YP, Gauthier L, Neumanna P (2012). Dead or alive: deformed wing virus and varroa destructor reduce the life span of winter honeybees. Appl Environ Microbiol.

[4] Traynor KS, Rennich K, Forsgren E, Rose R, Pettis J, Kunkel G, Madella S, Evans J, Lopez D, van Engelsdorp D (2016). Multiyear survey targeting disease incidence in US honey bees. Apidologie.

[5] Thaduri S, Stephan JG, de Miranda JR, Locke B. Disentangling host-parasite-pathogen interactions in a varroa-resistant honeybee population reveals virus tolerance as an independent, naturally adapted survival mechanism. Sci Rep. 2019;9(1). 10.1038/s41598-019-42741-6. 10.1038/s41598-019-42741-6 PMC6470206 30996279

[6] Locke B (2016). Natural Varroa mite-surviving Apis mellifera honeybee populations. Apidologie.

[7] Cornman RS, Tarpy DR, Chen Y, Jeffreys L, Lopez D, Pettis JS, et al. Pathogen webs in collapsing honey bee colonies. PLoS One. 2012;7(8). 10.1371/journal.pone.0043562. 10.1371/journal.pone.0043562 PMC3424165 22927991

[8] Ryabov EV, Childers AK, Chen Y, Madella S, Nessa A, VanEngelsdorp D, et al. Recent spread of Varroa destructor virus-1, a honey bee pathogen, in the United States. Sci Rep. 2017;7(1). 10.1038/s41598-017-17802-3. 10.1038/s41598-017-17802-3 PMC5727227 29234127

[9] Kim D, Langmead B, Salzberg SL (2015). HISAT: a fast spliced aligner with low memory requirements. Nat Methods.

[10] Dobin A, Davis CA, Schlesinger F, Drenkow J, Zaleski C, Jha S, Batut P, Chaisson M, Gingeras TR (2013). STAR: ultrafast universal RNA-seq aligner. Bioinformatics.

[11] Liao Y, Smyth GK, Shi W. featureCounts: an efficient general purpose program for assigning sequence reads to genomic features. Bioinformatics. 2014;30(7):923-30. 10.1093/bioinformatics/btt656. 10.1093/bioinformatics/btt656 24227677

[12] Pertea M, Pertea GM, Antonescu CM, Chang T-C, Mendell JT, Salzberg SL (2015). StringTie enables improved reconstruction of a transcriptome from RNA-seq reads. Nat Biotechnol.

[13] Harrow J, Frankish A, Gonzalez JM, Tapanari E, Diekhans M, Kokocinski F, Aken BL, Barrell D, Zadissa A, Searle S, Barnes I, Bignell A, Boychenko V, Hunt T, Kay M, Mukherjee G, Rajan J, Despacio-Reyes G, Saunders G, Steward C, Harte R, Lin M, Howald C, Tanzer A, Derrien T, Chrast J, Walters N, Balasubramanian S, Pei B, Tress M, Rodriguez JM, Ezkurdia I, van Baren J, Brent M, Haussler D, Kellis M, Valencia A, Reymond A, Gerstein M, Guigó R, Hubbard TJ. GENCODE: the reference human genome annotation for The ENCODE Project. Genome Res. 2012;22(9):1760-74. 10.1101/gr.135350.111. 10.1101/gr.135350.111 PMC3431492 22955987

[14] Langmead B, Salzberg SL (2012). Fast gapped-read alignment with bowtie 2. Nat Methods.

[15] Robinson MD, McCarthy DJ, Smyth GK. edgeR: a Bioconductor package for differential expression analysis of digital gene expression data. Bioinformatics. 2010;26(1):139-40. 10.1093/bioinformatics/btp616. 10.1093/bioinformatics/btp616 PMC2796818 19910308

[16] Love MI, Huber W, Anders S (2014). Moderated estimation of fold change and dispersion for RNA-seq data with DESeq2. Genome Biol.

[17] Elsik CG, Tayal A, Diesh CM, Unni DR, Emery ML, Nguyen HN, Hagen DE. Hymenoptera Genome Database: integrating genome annotations in HymenopteraMine. Nucleic Acids Res. 2016;44(D1):D793-800. 10.1093/nar/gkv1208. 10.1093/nar/gkv1208 PMC4702858 26578564

[18] Supek F, Bošnjak M, Škunca N, Šmuc T (2011). REVIGO summarizes and visualizes long lists of gene ontology terms. PLoS One.

[19] Bhella D (2015). The role of cellular adhesion molecules in virus attachment and entry. Philos Trans R Soc Lond Ser B Biol Sci.

[20] Maginnis MS (2018). Virus–receptor interactions: the key to cellular invasion. J Mol Biol.

[21] Rutter L, Carrillo-Tripp J, Bonning BC, Cook D, Toth AL, Dolezal AG (2019). Transcriptomic responses to diet quality and viral infection in Apis mellifera. BMC Genomics.

[22] Erban T, Sopko B, Kadlikova K, Talacko P, Harant K (2019). Varroa destructor parasitism has a greater effect on proteome changes than the deformed wing virus and activates TGF-β signaling pathways. Sci Rep.

[23] Whitfield CW, Behura SK, Berlocher SH, Clark AG, Johnston JS, Sheppard WS, Smith DR, Suarez AV, Weaver D, Tsutsui WD (2006). Thrice out of Africa: ancient and recent expansions of the honey bee, *Apis mellifera*. Science.

[24] Ryabov EV, Wood GR, Fannon JM, Moore JD, Bull JC, Chandler D, et al. A virulent strain of deformed wing virus (DWV) of honeybees (*Apis mellifera*) prevails after *Varroa destructor*-mediated, or in vitro, transmission. PLoS Pathog. 2014;10(6). 10.1371/journal.ppat.1004230. 10.1371/journal.ppat.1004230 PMC4072795 24968198

[25] Flenniken ML. Antiviral defense in invertebrates. Viruses. 2018;10(8). 10.3390/v10080403. 10.3390/v10080403 PMC6115728 30065149

[26] Doublet V, Poeschl Y, Gogol-Döring A, Alaux C, Annoscia D, Aurori C, Barribeau SM, Bedoya-Reina OC, Brown MJF, Bull JC, Flenniken ML, Galbraith DA, Genersch E, Gisder S, Grosse I, Holt HL, Hultmark D, Lattorff HMG, le Conte Y, Manfredini F, McMahon DP, Moritz RFA, Nazzi F, Niño EL, Nowick K, van Rij RP, Paxton RJ, Grozinger CM (2017). Unity in defence: honeybee workers exhibit conserved molecular responses to diverse pathogens. BMC Genomics.

[27] McMenamin AJ, Daughenbaugh KF, Flenniken ML. The Heat Shock Response in the Western Honey Bee (*Apis mellifera*) is Antiviral. Viruses. 2020;12(2). 10.3390/v12020245. 10.3390/v12020245 PMC7077298 32098425

[28] Traniello IM, Bukhari SA, Kevill J, Ahmed AC, Hamilton AR, Naeger NL, Schroeder DC, Robinson GE (2020). Meta-analysis of honey bee neurogenomic response links deformed wing virus type a to precocious behavioral maturation. Sci Rep.

[29] Rittschof CC, Rubin BER, Palmer JH (2019). The transcriptomic signature of low aggression in honey bees resembles a response to infection. BMC Genomics.

[30] Di Prisco G, Annoscia D, Margiotta M, Ferrara R, Varricchio P, Zanni V, Caprio E, Nazzi F, Pennacchio F (2016). A mutualistic symbiosis between a parasitic mite and a pathogenic virus undermines honey bee immunity and health. Proc Natl Acad Sci U S A.

[31] Galbraith DA, Yang X, Niño EL, Yi S, Grozinger C (2015). Parallel epigenomic and transcriptomic responses to viral infection in honey bees (Apis mellifera). PLoS Pathog.

[32] Brutscher LM, Daughenbaugh KF, Flenniken ML (2015). Antiviral defense mechanisms in honey bees. Curr Opin Insect Sci.

