# Supplementary material for: Multi-tiered analyses of honey bees that resist or succumb to parasitic mites and viruses
Source: BMC Genomics. 2021 Oct 6;22:720. doi: 10.1186/s12864-021-08032-z (PMC8493683; doi:10.1186/s12864-021-08032-z)
Supplement: Supplementary file 1 — Additional file 1 : Fig. S1. Heatmap of the sample-to-sample distance in R_mite v. R_control and S_mite v. S_control samples compiled using the R package DESeq2 with a customized map function, (http://bioconductor.org/packages/release/bioc/vignettes/DESeq2/inst/doc/DESeq2.html#heatmap-of-the-sample-to-sample-distances). Fig. S2. A) Principal Component Analysis of Gene Expression Data produced by DESeq 2 for R_mite, R_control, S_mite, and S_control. Note that PC1 captures 74% of the total variance between the samples, and shows the extreme divergence of R_mite from S_mite and S_control. PC2 provides differentiation of R_control from R_mite, revealing much of the gene expression differences attributable to mite infestation of the R genetic background. B) Principal Component Analysis of Differential Gene Expression data, contrasting R bees injected with virus, R_Virus, S bees injected with virus, S_Virus, and R & S bees injected only with a phosphate buffer solution, R_PBS and S_PBS; R_Virus and S_Virus are divergent in RNA sequencing data that comprise principal component 1; Principal component 2 captures the different responses to virus versus control injection in the R samples, and differences between the two S samples in response to buffer injection. Fig. S3. a) Scatterplot in semantic space of GO BP enrichment for UP in R_virus v. S_virus, FDR < 0.05 and logFC> 1.5. b) Scatterplot in semantic space of GO MF enrichment from HymenopteraMine set operation of Asymmetric Difference of UP in R_virus v. S_virus MINUS UP in R_virus v. R_PBS, genes with FDR < 0.05 and logFC > 1.5. Fig. S4. A) Mean Deformed wing virus levels separated by colony and by injection of viruses versus control (PBS). Diamond plot points indicate 95% confidence intervals B) Individual means and standard error estimates for each set of eight bees by colony and virus exposure. [file 12864_2021_8032_MOESM1_ESM.pdf]

Supplemental Figures

Figure S1. Heatmap of the sample-to-sample distance in R_mite v. R_control and S_mite v. S_control samples compiled using the R package DESeq2 with a customized map function, (http://bioconductor.org/packages/release/bioc/vignettes/DESeq2/inst/doc/DESeq2.html#heatmap-of-the-sample-to-sample-distances).

Figure S2. A) Principal Component Analysis of Gene Expression Data produced by DESeq 2 for R_mite, R_control, S_mite, and S_control. Note that PC1 captures 74% of the total variance between the samples, and shows the extreme divergence of R_mite from S_mite and S_control. PC2 provides differentiation of R_control from R_mite, revealing much of the gene expression differences attributable to mite infestation of the R genetic background. B) Principal Component Analysis of Differential Gene Expression data, contrasting R bees injected with virus, R_Virus, S bees injected with virus, S_Virus, and R & S bees injected only with a phosphate buffer solution, R_PBS and S_PBS; R_Virus and S_Virus are divergent in RNA sequencing data that comprise principal component 1; Principal component 2 captures the different responses to virus versus control injection in the R samples, and differences between the two S samples in response to buffer
injection.

Figure S3. a) Scatterplot in semantic space of GO BP enrichment for UP in R_virus v. S_virus, FDR<0.05 and logFC>1.5. b) Scatterplot in semantic space of GO MF enrichment from HymenopteraMine set operation of Asymmetric Difference of UP in R_virus v. S_virus MINUS UP in R_virus v. R_PBS, genes with FDR < 0.05 and logFC >1.5.

Figure S4. a) Deformed wing virus (DWV) loads as dCT, analyzed separately by ANOVA for each treatment (Control or Virus) in each colony. Comparison of means for each pair indicates two virus-injected samples which maintained very low virus levels, G2, and B5. B) ANOVA stats and means for each sample set.

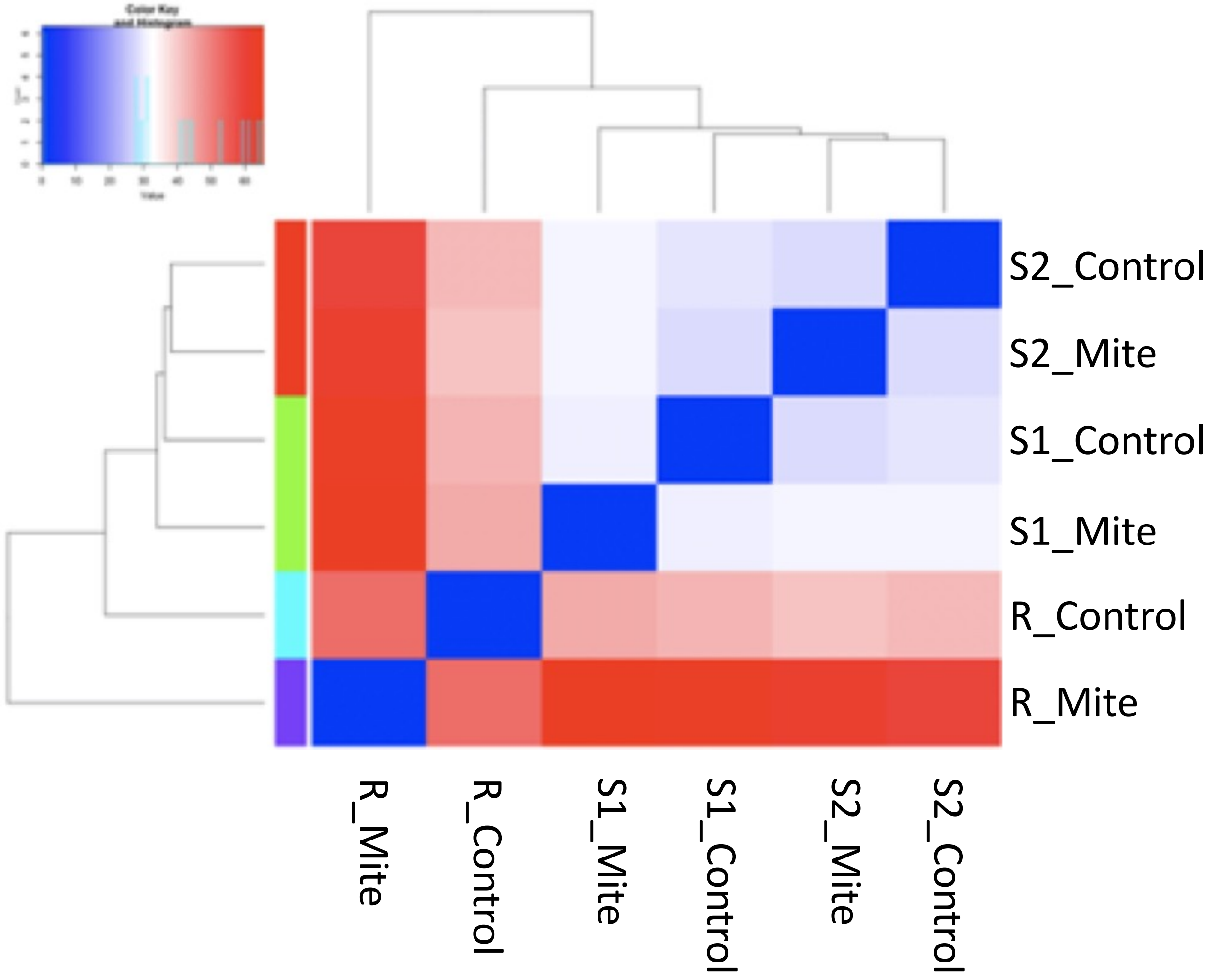
Color Key
and Histogram
Value
S2_Control
S2_Mite
S1_Control
S1_Mite
R_Control
R_Mite
R_Mite
R_Control
S1_Mite
S1_Control
S2_Mite
S2_Control

Figure S1

a)

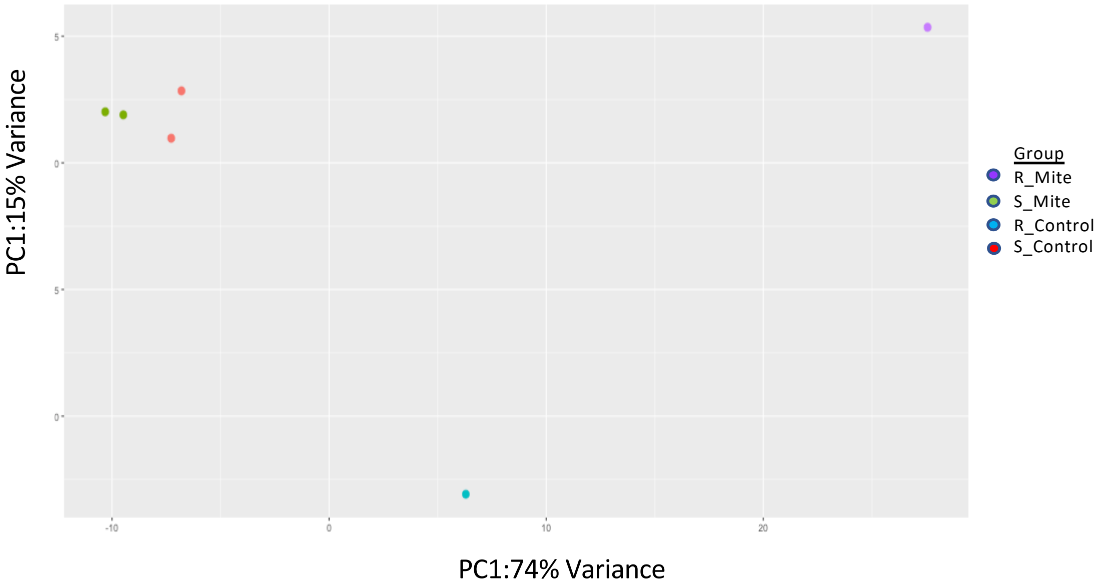
PC1:15% Variance
PC1:74% Variance
Group
R_Mite
S_Mite
R_Control
S_Control

b)

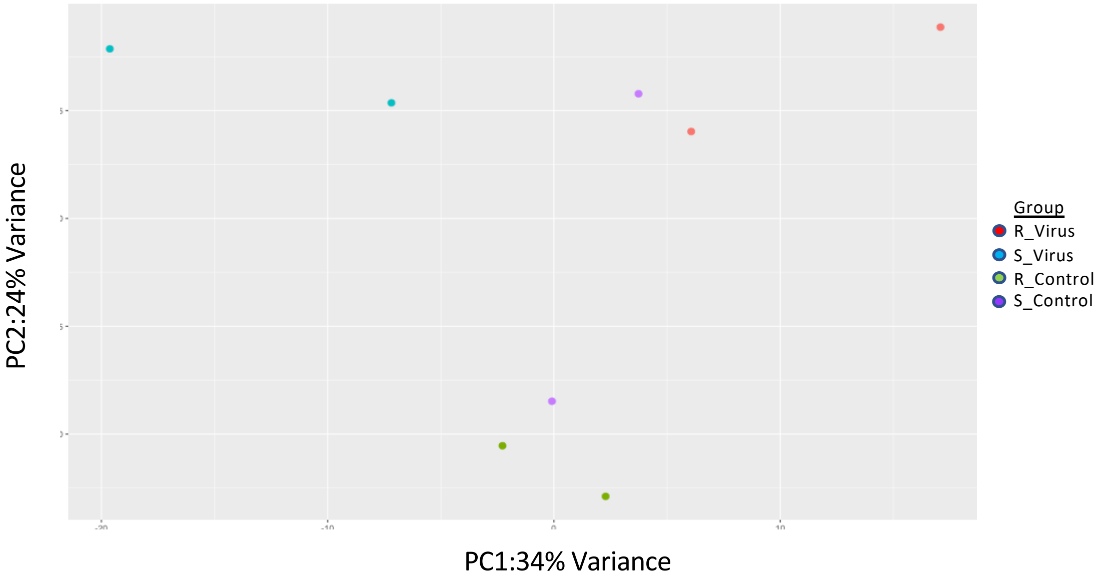
PC2:24% Variance
PC1:34% Variance
Group
R_Virus
S_Virus
R_Control
S_Control

Figure S2

a)

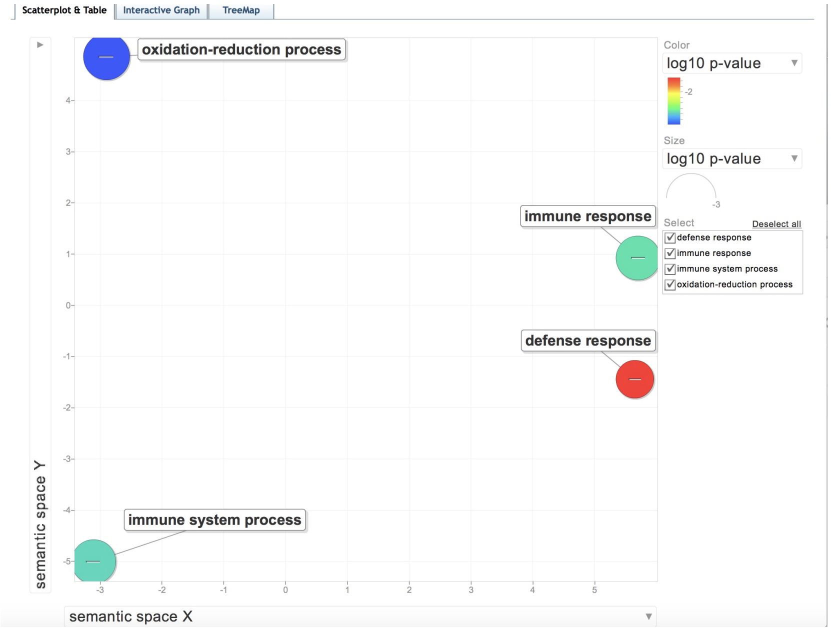
Scatterplot & Table
Interactive Graph
TreeMap
oxidation-reduction process
immune response
defense response
immune system process
semantic space Y
semantic space X
Color
log10 p-value
Size
log10 p-value
Select
Deselect all
defense response
immune response
immune system process
oxidation-reduction process

b)

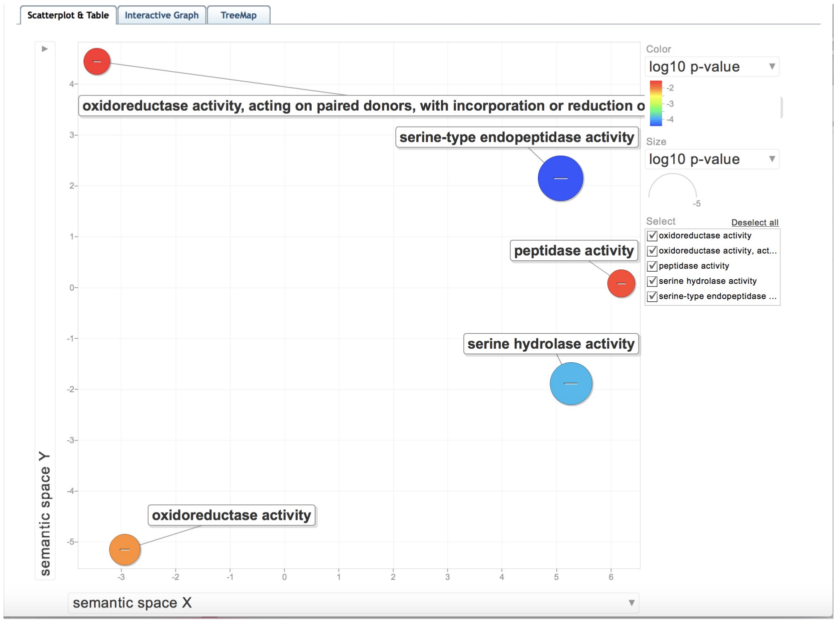
Scatterplot & Table
Interactive Graph
TreeMap
oxidoreductase activity, acting on paired donors, with incorporation or reduction o
serine-type endopeptidase activity
peptidase activity
serine hydrolase activity
oxidoreductase activity
semantic space Y
semantic space X
Color
log10 p-value
Size
log10 p-value
Select
Deselect all
oxidoreductase activity
oxidoreductase activity, act...
peptidase activity
serine hydrolase activity
serine-type endopeptidase ...

Figure S3

A)

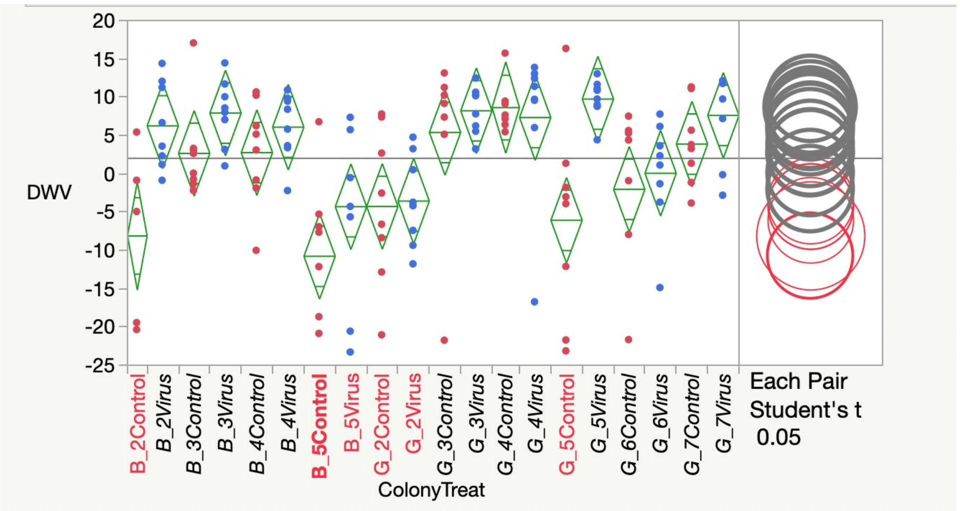

B)

Oneway Anova

Summary of Fit

Rsquare	0.394387
Adj Rsquare	0.309779
Root Mean Square Error	7.983688
Mean of Response	2.014231
Observations (or Sum Wgts)	156

Analysis of Variance

Source	DF	Sum of Squares	Mean Square	F Ratio	Prob > F
ColonyTreat	19	5645.125	297.112	4.6614	<.0001*
Error	136	8668.541	63.739		
C. Total	155	14313.666			

Means for Oneway Anova

Level	Number	Mean	Std Error	Lower 95%	Upper 95%
B_2Control	5	-8.122	3.5704	-15.18	-1.06
B_2Virus	8	6.244	2.8227	0.66	11.83
B_3Control	8	2.651	2.8227	-2.93	8.23
B_3Virus	8	7.920	2.8227	2.34	13.50
B_4Control	8	2.751	2.8227	-2.83	8.33
B_4Virus	8	6.079	2.8227	0.50	11.66
B_5Control	8	-10.781	2.8227	-16.36	-5.20
B_5Virus	8	-4.306	2.8227	-9.89	1.28
G_2Control	8	-4.274	2.8227	-9.86	1.31
G_2Virus	8	-3.574	2.8227	-9.16	2.01
G_3Control	8	5.388	2.8227	-0.19	10.97
G_3Virus	8	8.218	2.8227	2.64	13.80
G_4Control	7	8.629	3.0176	2.66	14.60
G_4Virus	8	7.334	2.8227	1.75	12.92
G_5Control	8	-6.084	2.8227	-11.67	-0.50
G_5Virus	8	9.744	2.8227	4.16	15.33
G_6Control	8	-2.038	2.8227	-7.62	3.54
G_6Virus	8	0.059	2.8227	-5.52	5.64
G_7Control	8	3.861	2.8227	-1.72	9.44
G_7Virus	8	7.613	2.8227	2.03	13.19

Std Error uses a pooled estimate of error variance
