# Supplementary material for: Multi-tiered analyses of honey bees that resist or succumb to parasitic mites and viruses
Source: BMC Genomics. 2021 Oct 6;22:720. doi: 10.1186/s12864-021-08032-z (PMC8493683; doi:10.1186/s12864-021-08032-z)
Supplement: Supplementary file 4 — Additional file 4 : Supplemental Table 3. Common genes identified in multiple studies that measured impacts of disease on honey bee gene expression. [file 12864_2021_8032_MOESM4_ESM.docx]

Table S1: Annotated list of genes differently regulated in the present study and in five recent RNASeq analyses of honey bee responses, {Rittschof, 2019 #13152;Doublet, 2017 #8536;Rutter, 2019 #14543;Traniello, 2020 #13138;McMenamin, 2020 #13137}. In last column, black font refers to our Experiment 2, blue font to Experiment 3.

| Accession | Name | Rittschoff Tables 2, 3 and 4 | Doublet | Rutter 10 genes from Table 1 virus main effects | Traniello 7 genes | McMenamin  & Brutscher | Current work |
| --- | --- | --- | --- | --- | --- | --- | --- |
| GB40148 | Cytochrome b561 domain-containing protein 2-like Y 100576555 | Up in low aggression  Up in immune activation | Up | N | N | Up in virus infection at 48 hours | Up  (R_virus v. S_virus)  (S_mite v. R_mite) &  (R_control v. R_mite) |
| GB40212 | Protein mesh N 725498 | Down in immune activation  Down in low aggression | Down | N | N | Down in virus infection at 48 hours | Up  (R_virus v. S_virus)  Down  (S_virus v. S_PBS)  (R_mite v. S_mite)  (R_mite v. R_control)  (S_mite v. S_control) |
| GB40261 | GILT-like protein 1  [552600](http://128.206.116.3:8080/hymenopteramine/report.do?id=80513751) | N |  | N | N | Down in virus infection at  48 & 72 hours | Up  (R_virus v. S_virus)  (S_control v. R_mite)  (S_mite v. R_mite) |
| GB40696 | Mast cell degranulating peptide  [406134](http://128.206.116.3:8080/hymenopteramine/report.do?id=80227595) | N |  | N | N | N | Up  (R_virus v. S_virus)  (S_control v. R_control)  (S_control v. R_mite)  (S_mite v. R_control)  Down  (S_virus v. S_PBS) |
| GB40697 | Apamin, neurotoxin  [406135](http://128.206.116.3:8080/hymenopteramine/report.do?id=80227609) | N |  | N | N | N | Up  (R_control v. R_mite)  (S_mite v. R_mite)  (S_control v. R_control)  (S_control v. R_mite)  Down  (R_control v. S_mite) |
|  |  |  |  |  |  |  |  |
| GB41361 | Cytochrome b5-like  724654 | Up in immune activation  Up in low aggression | Up | N | N | N | Up  (R_virus v. S_virus)  (R_control v. R_mite)  Down  (S_virus v. S_PBS) |
| GB41428 | Def-1  406143 | Up in immune activation  Up in low aggression | Up | N | N | N | Up (R_virus v. S_virus)  (S_mite v. R_mite)  (S_control v. R_mite)  (R_control v. R_mite)  Down  (S_virus v. S_PBS)  (R_control v. S_mite) |
| GB41483 | krueppel | N |  | N | N | N | Down (R_virus v. S_virus) |
| GB41545 | MD-2-related lipid recognition protein  [409187](http://128.206.116.3:8080/hymenopteramine/report.do?id=80257381) | N |  | Over-represented in non-virus group | N | N | Up  (R_virus v. S_virus)  (S_mite v. R_mite)  (R_control v. R_mite)  Down  (S_virus v. S_PBS)  (S_virus v. R_PBS) |
| GB42472 | Dorsal 2  [411712](http://128.206.116.3:8080/hymenopteramine/report.do?id=80526992) |  |  |  |  | N | UP  (S_virus v. R_PBS)  Down  (R_virus v. S_virus) |
| GB42598 | tetrapeptide  repeat associated homeobox protein 1-like  [100578625](http://128.206.116.3:8080/hymenopteramine/report.do?id=80599855) | N |  | N | N | Down in virus infection at 6 hours | Up  (S_mite v. R_mite)  (S_control v. R_mite)  Down  (R_control v. S_mite) |
| GB42692 | Ultraspiracle  [409227](http://128.206.116.3:8080/hymenopteramine/report.do?id=80594501) | N |  | N | N | Up in virus infection at 6 hours | Down  (R_virus v. S_virus)  Up  (R_control v. R_mite) |
| GB42797 | Circadian clock-controlled protein 726981 | Down in immune activation  Down in low aggression | Down | N | N | Down in virus infection at 6 hours | Down (S_virus v. S_PBS)  (S_virus v. R_PBS) &  (S_mite v. R_mite) |
| GB43006 | Glucose dehydrogenase [FAD, quinone]  408603 | Down in immune activation  Down in low aggression | Down | N | N | N | Up (R_virus v. S_virus)  Down  (S_mite v. R_mite),  (S_virus v. S_PBS)  (S_mite v. R_control) |
| GB43456 | 18-wheeler [410902](http://128.206.116.3:8080/hymenopteramine/report.do?id=80333979) |  |  |  |  | N | UP  (R_control v. R_mite)  (S_control v. R_mite)  (S_mite v. R_mite) |
|  |  |  |  |  |  |  |  |
| GB43738 | PPO Prophenoloxidase  [406155](http://128.206.116.3:8080/hymenopteramine/report.do?id=80266161) |  |  |  |  | Up in virus infection @48 hours  Down in virus infection @ 6 hours | Up  (R_virus v. S_virus)  Down  (S_virus v. S_PBS)  (S_virus v. R_PBS) |
| GB43823 | Chemosensory protein 1 Y 725382 | Down by immune activation  Up in low aggression | Down | N | N | Down in virus infection at 72 hours | Down  (S_mite v. R_control)  (S_control v. R_control)  (S_control v. R_mite)  (S_mite v. R_mite) |
|  |  |  |  |  |  |  |  |
| GB44112 | Melittin  [406130](http://128.206.116.3:8080/hymenopteramine/report.do?id=80445393) | N |  | N | N | N | Up (R_virus v. S_virus)  (S_mite v. R_mite)  Down  (S_virus v. S_PBS)  (R_PBS v. S_PBS)  (S_virus v. R_PBS) |
|  |  |  |  |  |  |  |  |
| GB44367 | Phospholipase A2-like [724436](http://128.206.116.3:8080/hymenopteramine/report.do?id=80530640) | N |  | N | N | N | Up  (R_virus v. S_virus)  (R_virus v. R_PBS)  Down  (S_virus v. S_PBS)  (R_PBS v. S_PBS) |
| GB44824 | Corazonin receptor Y 409042 | Up in immune activation and low aggression | Up | N | N | N | Down (S_virus v. S_PBS) |
| GB44996 | Hexamerin 110  [551648](http://128.206.116.3:8080/hymenopteramine/report.do?id=80202149) |  |  |  |  | Down in virus infection at 48 hours | Up  (R_control v. R_mite)  (S_control v. R_mite)  (S_virus v. R_PBS)  Down  (R_virus v. S_virus)  (R_virus v. S_PBS)  (R_PBS v. S_PBS) |
| GB45499 | Sodium-coupled monocarboxylate transporter 2 N 410683 | Down in immune activation and low aggression | Down | N | N | N | Up  (S_mite v. R_mite)  (R_virus v. S_virus)  (S_control v. R_mite) |
| GB45909 | ([724274](http://128.206.116.3:8080/hymenopteramine/report.do?id=80334892)) protein lethal(2) essential for life homolog 1 |  |  |  |  | N | Down  (S_control v. R_control)  (724274) |
| GB45910 | ([724367](http://128.206.116.3:8080/hymenopteramine/report.do?id=80334900)) protein lethal(2) essential for life homolog 2 |  |  |  |  | Up in virus infection at 72 hours | Up  (R_control v. R_mite)  (S_control v. R_mite)  (S_mite v. R_mite)  (S_mite v. R_control)  Down  (S_control v. S_mite) |
| GB46223 | Odorant binding protein 14 [677673](http://128.206.116.3:8080/hymenopteramine/report.do?id=80288604) | Down in immune activation and low aggression | Down | N | N | Up in virus infection at 72 hours | Up  (R_virus v. S_virus)  Down  (S_virus v. S_PBS)  (S_virus v. R_PBS)  (R_PBS v. S_PBS) |
| GB47546 | Apidaecin 1  [406140](http://128.206.116.3:8080/hymenopteramine/report.do?id=80319300) | N | Up | N | Up in DWV | Up in virus infection at 72 hours | Up  (R_virus v. S_virus)  (R_virus v. R_PBS)  (R_virus v. S_PBS)  (S_mite v. R_mite)  (S_control v. R_mite)  (R_control v. R_mite)  (R_control v. S_control) |
| GB47618 | Def-2  413397 | Up in immune activation and low aggression | Up |  |  | N | Up  (R_virus v. S_PBS) |
| GB47804 | Peptidoglycan recognition protein s1  [725158](http://128.206.116.3:8080/hymenopteramine/report.do?id=80253644) | N | Inconsistent and variable | N | N | N | Up  (R_virus v. S_virus)  (R_virus v. R_PBS)  (R_virus v. S_PBS)  (S_mite v. R_mite)  (S_control v. R_mite) |
| GB47805 | Peptidoglycan recognition protein  s2  [412484](http://128.206.116.3:8080/hymenopteramine/report.do?id=80253656) | N | Inconsistent and variable | N | Up in DWV | Up in virus infection @ 6 & 48 hours | Up  (R_virus v. S_virus)  (S_mite v. R_mite)  (R_control v. R_mite)  Down  (S_virus v. R_PBS) |
| GB47880 | Superoxide dismutase 1 [409398](http://128.206.116.3:8080/hymenopteramine/report.do?id=80537497) | N |  | N | N | N | Up  (S_mite v. R_mite)  ((S_mite v. R_control)  (S_control v. R_control)  (S_control v. R_mite) |
| GB47974 | Carboxylesterase  726134 | Down in immune activation and low aggression | Down | N | N | N | Up  (R_virus v. S_virus)  Down  (S_virus v. R_PBS) |
| GB48134 | Lactate dehydrogenase-like  411188 | Up in immune activation and low aggression | Up – hub gene with many connections to other DE genes | N | N | N | Up  (S_mite v. R_mite)  (S_control v. R_mite)  Down  (S_control v. S_mite)  (R_control v. S_mite) |
| GB48228 | Phospholipase A2  [406141](http://128.206.116.3:8080/hymenopteramine/report.do?id=80245338) | N |  | N | N | N | Up  (R_virus v. S_virus)  (S_mite v. R_mite)  (S_control v. R_mite)  (R_control v. R_mite)  Down  (S_virus v. S_PBS)  (R_virus v. R_PBS)  (S_virus v. R_PBS)  and Erban DWV |
| GB48505 | chitinase like protein 4  [413324](http://128.206.116.3:8080/hymenopteramine/report.do?id=80569292) |  |  |  | Up in DWV  ? ambiguous GB ID | Up in virus infection at 48 hours ?  ambiguous GB ID | Not DE |
| GB52829 | chitinase like protein 4  [413324](http://128.206.116.3:8080/hymenopteramine/report.do?id=80569292) | N |  | N | Up in DWV  ?  Ambiguous  GB ID | Up in virus infection at 48 hours  ?  ambiguous GB ID | Up  (R_control v. R_mite)  (S_mite v. R_mite)  (S_control v. R_mite) |
| GB48923 | dicer  [726766](http://128.206.116.3:8080/hymenopteramine/report.do?id=80132586) |  |  |  |  | Up in virus infection at 72 hours | Up  (R_mite v. R_control) |
| GB49544 | Vitellogenin 406088 | Down in immune activation and low aggression | Down | N | N | Up in virus infection at 6 hours; Down at 48 hours | Up  (R_virus v. S_virus)  (R_PBS v. S_PBS)  (S_mite v. R_mite)  (R_control v. R_mite)  Down  (S_virus v. S_PBS) |
| GB49709 | Coiled-coil domain-containing protein 86 551400 | Up in immune activation  Down in low aggression | Up | N | N | N | Down  (S_mite v. R_mite)  (S_mite v. R_control) |
| GB50116 | chymotrypsin inhibitor  [725202](http://128.206.116.3:8080/hymenopteramine/report.do?id=80298506) | Brain | Down | N |  | Down in virus infection at 72 hours | Up  (R_virus v. S_virus)  (S_mite v. R_mite)  (S_control v. R_control)  (S_control v. R_mite)  Down (R_control v. S_mite) |
| GB50218 | Ornithine aminotransferase, mitochondrial 410583 | Down in immune activation and low aggression |  | N | N | Up in virus infection at 48 hours  Down in virus infection at 72 hours | Up  (R_virus v. S_virus)  (S_mite v. R_mite)  Down  (S_virus v. R_PBS) |
| GB50423 | IRP30  [408807](http://128.206.116.3:8080/hymenopteramine/report.do?id=80429862)  Immune response protein | Down in immune activation  Up in low aggression |  | N | N | Up in virus infection at 6 hours | Up  (R_virus v. S_virus)  (S_mite v. R_mite)  (R_control v. R_mite)  Down  (S_control v. S_mite)  (R_control v. S_mite) |
| GB50955 | Argonaute-2  [411577](http://128.206.116.3:8080/hymenopteramine/report.do?id=80168156) |  |  |  |  | Up in virus infection at 48 & 72 hours | Up  (R_virus v. S_virus)  Down  (S_control v. R_control) |
| GB51223 | Hymenoptaecin Y 406142 | Up & Down in immune activation  Up in low aggression | Up/Down | N | N | Up in virus infection at 6 & 48 hours; Down after 72 | Up  (R_virus v. S_virus)  (S_mite v. R_mite)  (S_control v. R_mite)  (R_control v. R_mite)  Down  (R_control v. S_mite)  (S_control v. S_mite) |
| GB51498 | MyD88  [413194](http://128.206.116.3:8080/hymenopteramine/report.do?id=80392384) |  |  |  |  | Down in virus infection @ 48 hours | UP (R_mite v. R_control)  (R_mite v. S_control) |
| GB52023 | Cytochrome P450 6AQ1  408383 | Down in immune activation and low aggression | Down | N | N | N | Up  (R_virus v. S_virus)  (R_PBS v. S_PBS)  Down  (S_virus v. R_PBS) |
|  |  |  |  |  |  |  |  |
| GB53110 | Apidermin 3  [409716](http://128.206.116.3:8080/hymenopteramine/report.do?id=80420297) | N | DE but inconsistent and variable | N | N | N | Up  (R_PBS v. S_virus)  (S_mite v. R_mite)  (R_control v. R_mite)  (S_control v. R_control)  (S_control v. R_mite)  Down  (R_virus v. S_virus)  (S_virus v. S_PBS)  (R_virus v. R_PBS)  (S_virus v. R_PBS)  (R_PBS v. S_PBS)  (R_control v. S_mite) |
| GB53302 | cactus 3; mini-chromosome maintenance complex binding protein  [552778](http://128.206.116.3:8080/hymenopteramine/report.do?id=80602701) |  |  |  |  | N | UP (R_mite v. R_control) |
| GB54097 | Malvolio Y 494509 | Up in immune activation and low agression | Up | N | N | N | Up  (R_virus v. S_virus)  (S_mite v. R_mite)  Down  (S_virus v. S_PBS)  (S_virus v. R_PBS) |
| GB54506 | Sushi domain containing membrane protein with scavenger receptor activity  [411253](http://128.206.116.3:8080/hymenopteramine/report.do?id=80553338) | N |  | N | N | Up in virus infection at 48 hours | Up  (R_virus v. S_virus)  (S_mite v. R_mite)  (S_control v. R_mite)  Down  (S_virus v. S_PBS)  (S_virus v. R_PBS) |
| GB54571 | FACT complex subunit Ssrp1 N 726058 | Up in immune activation  Down in low aggression | Up | N | N | N | Up  (R_mite v. S_mite)  Down  (S_mite v. R_mite)  (S_control v. R_mite)  (S_mite v. R_control) |
| GB54774 | armadillo  [408399](http://128.206.116.3:8080/hymenopteramine/report.do?id=80021669) | N |  | N | N |  | Up  (S_mite v. R_mite)  (R_control v. R_mite)  (S_control v. R_mite) |
| GB55204 | Major royal jelly protein 3  [406121](http://128.206.116.3:8080/hymenopteramine/report.do?id=80181432) | N |  | N | N | Up in virus infection at 6 hours | Down  (R_control v. R_mite) |
| GB55205 | Major royal jelly protein 1  [406090](http://128.206.116.3:8080/hymenopteramine/report.do?id=80181456) | N | Down | N | N | N | Up  (S_control v. R_control)  (S_virus v. R_PBS)  Down  (R_control v. R_mite)  (R_control v. S_mite) |
| GB55212 | Major royal jelly protein 2  [406091](http://128.206.116.3:8080/hymenopteramine/report.do?id=80181582) | N |  | N | N | N | Down (S_virus v. R_PBS) |
| GB42218 | Acyl-CoA delta 11 desaturase ([724226](http://128.206.116.3:8080/hymenopteramine/report.do?id=80415295);  [100576797](http://128.206.116.3:8080/hymenopteramine/report.do?id=80045503) -  ambiguous) |  |  |  | Up with DWV – possibly ambiguous GB ID | Down in virus infection at 72 hours (724226) | Up  (R_virus v. S_virus)  (R_virus v. R_PBS)  Down  (S_virus v. S_PBS)  (S_mite v. R_mite)  (R_control v. R_mite)  (S_control v. R_mite) |
| GB51238 | Acyl-CoA delta 11 desaturase  [551527](http://128.206.116.3:8080/hymenopteramine/report.do?id=80415411) |  |  |  | Up with DWV – possibly ambiguous GB ID | Up in virus infection at 48 hours | Up  (R_virus v. S_virus)  (R_virus v. S_PBS) |
| GB55701 | Aldh family 7 member  A1 homolog  [411140](http://128.206.116.3:8080/hymenopteramine/report.do?id=80512552) |  |  |  | Up with DWV | N | Up  (R_virus v. S_virus)  Down  (S_virus v. S_PBS)  (S_virus v. R_PBS) |
| GB44455 | Homolog of D.m. *pirk;*  LOC100578156 | N | Co-expressed with PGRP-S2 | N | N | Up in virus infection at 72 hours | Up  (R_virus v. S_virus)  (R_PBS v. S_virus)  (S_mite v. R_mite)  Down  (S_control v. R_control)  (S_control v. S_mite) |
