# Supplementary material for: Multi-tiered analyses of honey bees that resist or succumb to parasitic mites and viruses
Source: BMC Genomics. 2021 Oct 6;22:720. doi: 10.1186/s12864-021-08032-z (PMC8493683; doi:10.1186/s12864-021-08032-z)
Supplement: Supplementary file 5 — Additional file 5 : Supplemental Text 1. Additional textual highlights of our results regarding individual genes found associated with honey bee mite and virus interactions in prior work. [file 12864_2021_8032_MOESM5_ESM.docx]

Supplemental Text 1.

Additional textual highlights of our results regarding individual genes found associated with honey bee mite and virus interactions in prior work.

GB47805 (Pgrp-2), or peptidoglycan recognition protein 2, is associated with DWV levels in our experiments and uniformly associated with DWV presence in honey bee brains of DWV infected bees {Traniello, 2020 #13138}. GB47805 was earlier found to be up-regulated in CCD bees {Johnson, 2009 #2620} and consistently present in transcriptome datasets of pathogen-associated gene expression {Doublet, 2017 #13136}. We find Pgrp-2 (GB47805) UP in R_virus v. S_virus and UP in S_mite v. R_mite - where DWV infection levels are higher in S_mite. GB47805 is also DOWN in S_virus v. R_Control; UP in R_control v R_mite; and UP in R_virus v. S_Control. Our results suggest that elevated expression of GB47805 may be a marker of DWV infection intensity or anti-viral activity, and Pgrp-2 expression distinguishes S bees injected with virus from R bees injected with virus - DOWN in S_virus v. R_Control but UP in the R_virus v. S_Control contrast. Our results are also consistent with up-regulation of GB47805 in the R genetic background and correlated with the DWV resistance of R bees.

Traniello et al. {Traniello, 2020 #13138} identify Acyl-CoA delta 11 desaturase as one of the 7 genes consistently differentially expressed (DE) in their brain transcriptomes and associated with DWV. However, there are multiple honey bee gene IDs in BeeBase and RefSeq that are annotated as Acyl-CoA delta 11 desaturase, and it is not clear to which Traniello et al. refer. Accordingly, we report on all of them. GB42218 (100576797 and 724226; ambiguous) is UP in R_virus v. S_virus, UP in R_virus v. R_Control and DOWN in S_virus v S_Control. GB42218 is also DOWN in S_mite v. R_mite, DOWN in R_control v. R_mite, and DOWN in S_mite v. R_mite. Confusingly, GB51236 (552417) and GB51238 (551527) are also identified as Acyl-CoA delta 11 desaturase. GB51238 is UP in R_virus v. S_virus, UP in R_virus v. S_Control, and one of the 164 genes UP in the Asymmetric Difference of UP in R_virus v. S_virus MINUS UP in R_virus v. R_Control, as well as one of the 185 genes UP in the Asymmetric Difference of UP in R_virus v S_virus MINUS UP in R_Control v. S_Control. This pattern of expression is consistent with GB51238 and GB42218 being turned up by DWV, suppressed by Varroa, and elevated generally in R bees compared to S bees. Moreover, differentially elevated expression of GB51238 and GB42218 associates with the enhanced viral resistance of R bees over S bees, and is even more convincingly associated with viral resistance after subtracting gene expression differences occurring in R bees and S bees injected with PBS. GB51236, on the other hand, is not DE in any sample comparison we examined.

Apidaecin (GB47546) is an antimicrobial peptide, associated with viral infection and antiviral response in multiple studies {Doublet, 2017 #8536}, {Traniello, 2020 #13138}, that was found frequently in our DE gene lists as well. GB47546 is UP in R_virus v. S_virus; UP in R_virus v. S_Control; UP in R_virus v. R_Control; UP in S_mite v. R_mite; DOWN in S_mite v. R_control; UP in S_control v. R_mite; and DOWN in S_control v. R_control. Accordingly, our results show elevated expression of Apidaecin is usually elicited by DWV infection, but the expression level is not correlated perfectly with DWV load (e.g., DOWN in S_mite v. R_control & DOWN in S_control v. R_control - despite DWV levels being higher in S_mite and S_control than in R_control). Once again this antiviral response appears to be confounded by Varroa mite infestation or genetic heritage or both. For example, GB47546 is DOWN in S_mite v. R_control and DOWN in S_control v. R_control; S_control has higher DWV levels than both R_control and R_mite.

Idgf4 - chitinase like protein 4 (GB48505; GB52829) was reported as one of 6 genes positively associated with DWV infection in an analysis of previous studies by Traniello et al. {Traniello, 2020 #13138}. GB52829 is differentially expressed in Experiment 2, and UP in S_mite v. R_mite; UP in S_control v. R_mite; UP in R_control v R_mite. Our results support the positive association between Idfg4 and DWV in the S_mite v. R_mite contrast and the S_control v. R_mite contrast because the S bees have higher DWV infection levels. The elevated expression of Idgf4 in R_control v. R_mite suggests expression of GB52829 may also be reduced by mite infestation, though R_control has marginally higher DWV loads than R_mite. Idgf4 did not appear differentially expressed after DWV injection.

We note that we have DE evidence for 6 of the 7 genes that identify as associated with DWV in the brain transcriptome datasets present in their meta-analysis - the exception being GB51444 (724619). Vitellogenin (GB49544) expression data in our experiments is in partial accord with what has been reported in earlier studies, where Vg appears down-regulated by pathogens generally (See e.g., Doublet, et al, 2017 {Doublet, 2017 #8536}). GB49544 may also be expressed at higher levels by R bees than S bees, and suppressed by Varroa mites. Vitellogenin is UP in R_virus v. S_virus (with higher levels of DWV in S_virus), UP in R_Control v S_Control (higher expression in R ), DOWN in S_virus v. R_Control, UP in S_mite v. R_mite (higher DWV load in S_mite than R_mite), and UP in R_control v. R_mite.

GB41545 (LOC409187), or MD-2-related lipid recognition protein involved in cholesterol transport, has been implicated in anti-viral defenses in honey bees {Rutter, 2019 #14543}. GB41545 is UP in R_virus v. S_virus, UP in S_mite v. R_mite, DOWN in S_virus v. R_Control, UP in S_control v. R_mite and UP in R_control v. R_mite. Our results are partially consistent with GB41545 expression being induced by viral infection and part of an anti-viral response, but not completely - (c.f., DOWN in S_virus v. R_Control; and DOWN in S_virus v. S_Control) so genetic differences between S and R bees may play a role in the expression levels of GB41545. GB41545 expression may also be suppressed by Varroa mite parasitism.

Genomic components of the RISC complex such as Dicer and Argonaute have been positively associated with DWV infection {Galbraith, 2015 #6849;Brutscher, 2015 #6565}. We find that GB48923, (726776) or Dicer, is DOWN in R_control v. R_mite - equivalent to increased expression in R_mite compared to R_control. GB50955 (LOC411577) or Argonaute-2 is listed as one of 10 DE genes associated with virus in Table 1 of Rutter, 2019 {Rutter, 2019 #14543}. However, GB50995 is UP in R_virus v. S_virus, where S_virus bees have higher DWV loads, and DOWN in S_control v. R_control, where S_control bees have higher levels of DWV. Our evidence leads equally well to Argonaute-2 expression being associated with elevated expression in DWV and Varroa -resistant R bees and supports the possibility that Argonaute-2 expression could be associated with viral resistance mechanisms in R bees.

Toll 6 receptor (LOC410229), or GB48396, was one of the most differentially expressed genes in a study of transcriptomic responses to viral infection {Galbraith, 2015 #6849}, but it does not occur in any list of differentially expressed genes in the contrasts we examined in this report. However, another Toll-6 like receptor, Tollo, GB48426 (LOC410235) was UP in S_mite v. R_mite, UP in S_control v. R_mite and UP in R_control v. R_mite. Thus, GB48426 is positively correlated with higher DWV levels in S_mite and S_control and marginally higher DWV levels in R_control, though genetic and phenological differences between R and S cannot be excluded, either. Rutter et al., 2019 {Rutter, 2019 #14543}also identify GB47407, or Histone H-4 as one of 10 mapped genes differentially expressed in the virus-effect arm of their study. We find GB47407 in several DE lists (UP in S_mite v. R_mite; DOWN in R_virus v. S_virus; DOWN in R_Control v. S_virus; UP in S_control v R_control - (where S_mite, S_virus and S_control all have higher DWV levels than the other sample), generally confirming Histone H4 may be associated with antiviral defenses in these contrasts. Tropomyosin, or GB51305 (LOC551252) is another gene suggested to be associated with DWV infection by Rutter, et al.{Rutter, 2019 #14543}, but we find GB51305 differentially expressed in only one sample comparison, and is UP in R_virus v S_virus.

The other genes identified by Rutter et al. {Rutter, 2019 #14543}, and listed in their Table 1 of 10 mapped genes differentially expressed in the virus main-effects of their study, were not present in any of our lists of differentially expressed genes in the various sample contrasts we explored. For instance, GB48755, GB50178, GB42313, GB53500, GB54503 and GB50813 are not DE in any of our lists. GB55701 (LOC411140) an aldehyde dehydrogenase homolog associated with DWV infection in Traniello, I.M., 2020 {Traniello, 2020 #13138}appears in the differentially expressed genes in Experiment 3 and is UP in R_virus v. S_virus; but DOWN in S_virus v. R_Control. Our results are consistent with Traniello, et al., only if R bees express GB55701 at higher levels than S bees, including S bees injected with DWV compared to R bees injected with PBS.

GB54315, a protein with acyl-coA transferase activity, is found in several of our lists, including UP in R_virus v. S_virus. Consistent with this result, Traniello et al. {Traniello, 2020 #13138} identified it as negatively associated with DWV infection. McMenamin et al. {McMenamin, 2020 #13137},provide evidence that heat shock proteins are involved in the antiviral response in honey bees, using recombinant Sindbis virus (SINV-GFP) as a surrogate, demonstrating that expression of a hsp20 homolog, aka protein lethal(2) essential for life, is an important antiviral response. But there are at least five homologs or paralogs in honey bee ([LOC410857](http://128.206.116.3:8080/hymenopteramine/report.do?id=80346645), [LOC412197](http://128.206.116.3:8080/hymenopteramine/report.do?id=80122636), [LOC724367](http://128.206.116.3:8080/hymenopteramine/report.do?id=80334900), [LOC724449](http://128.206.116.3:8080/hymenopteramine/report.do?id=80334916), [724274)](http://128.206.116.3:8080/hymenopteramine/report.do?id=80334892) and two lethal(2) essential for life homologs DE in our results. The first, GB45909 ([724274](http://128.206.116.3:8080/hymenopteramine/report.do?id=80334892))), is differentially expressed and DOWN in S_control v. R_control. But the second, GB45910 ([724367](http://128.206.116.3:8080/hymenopteramine/report.do?id=80334900)) is UP in S_mite v. R_mite, UP in R_control v. R_mite, UP in S_control v. R_mite, but DOWN in S_control v. S_mite and DOWN in R_control v. S_mite, and thus correlated with DWV levels. None of the others are DE in any list of ours. McMenamin et al. {McMenamin, 2020 #13137} also show that GB51659 (552223) dnaJ homolog shv, Hsp90 (GB40976; 408928), Hsp83 (GB45495, 411700), and Hsc70-3 and Hsc70-4 are all involved in the antiviral response to SINV-GFP. We find only Hsp70-4, or GB40866 (409418), also known as Hsp70-c, differentially expressed in our work. GB40866 is UP in S_mite v. R_mite and UP in S_control v. R_mite, where S_mite and S_control have the higher DWV titers in these contrasts. So, our results support increased Hsp70-c (GB40866) expression as an antiviral response and associated with DWV infection.

Rittschoff et al. {Rittschof, 2019 #13152} found that many genes differentially expressed in “low-aggression” bees are also affected by pathogen infection and parasite feeding, concluding that the molecular signature of low aggression resembles a diseased state. Rittschof Table 2 provides a list of 13 genes that significantly overlap genes up-regulated by pathogens or parasites in Doublet et al., 2017 {Doublet, 2017 #8536}, while Rittschof et al. Table 3 shows non-significant overlap with genes down-regulated in Doublet. Some of the Rittschof et al. Table 2 genes (characterized as up-regulated by immune activation) are differentially expressed in our experiments, but others are not. We also find significant differential expression of two genes from Rittschof et al. Table 3, Vitellogenin and Hymenoptaecin - but many other genes, both up- and down-regulated in Doublet et al. and listed in Rittschof et al. Tables 2 & 3 are not DE in our experiments. Additionally, Erban, et al. {Erban, 2019 #13131}, describe proteomic perturbations associated with Varroa mites and DWV that bear brief review and comparison with our results. Among the peptides showing the largest changes are Pla2, that we also find DE in key experimental contrasts of R and S bees affected by mites and virus. Erban et al. suggest that Pla2 GB48228 protein production is down-regulated by both Varroa and DWV. We find Pla2 is UP in R_virus v. S_virus, and UP in S_mite v. R_mite, UP in S_control v. R_mite, and UP in R_control v. R_mite and also differentially expressed in other key contrasts from our work: e.g., extremely down-regulated in S_virus v. R_Control, DOWN in S_virus v. S_Control, and DOWN in R_Control v. S_Control. These results support Pla2 expression being down-regulated by DWV injection in S bees, but Pla2 expression elevated in R bees after virus injection, thus corresponding to heightened DWV resistance and lower DWV loads in R bees. In further support, Pla2 is UP in S_control v. R_mite, and UP in R_control v. R_mite, remembering that DWV levels are higher in S_control than R_mite. Erban, et al. {Erban, 2019 #13131} also identify a polypeptide XP_003249944.1, described as tetrapeptide repeat associated homeobox protein 1-like. They find this protein is highly elevated by both DWV and Varroa. We ascertained that the corresponding gene is GB42598 (LOC100578625). GB42598 expression is DOWN in S_mite v. R_control, UP in S_control v. R_mite, UP in S_mite v. R_mite, and thus expression appears suppressed by mites, but suppressed less in the S than R heritage when mites are present.

Even when GO enrichment results are limited it is possible to glean other distinctive differences between R and S bees and their respective response to mites and DWV. For instance, the 51 genes UP in R_virus v. S_virus and UP in S_mite v. R_mite included not only Apidaecin GB47546, Defensin-1, GB41428 , Hymenoptaecin GB51223 and a gamma interferon inducible thiol reductase like protein (GILT-1), GB40261, but also genes encoding Vitellogenin GB49544 and Malvolio GB54097, the venom components Mellitin-2 GB44112, Phospholipase A-2 GB48228 , a venom serine protease GB44120 and Apamin (GB40697) a neurotoxin. The peptidoglycan recognition proteins GB47805 and GB47804, a cell matrix adhesion molecule, GB40210, various cellular membrane components and transmembrane transporters, e.g., GB50447, lipid recognition proteins, e.g., GB41545 a MD-2-related lipid recognition protein previously implicated in antiviral defenses in honey bees, and involved in cholesterol transport, a protein with DNA transcription factor activity GB52620, a protein with scavenger receptor activity GB54506, other cholesterol and lipid transport proteins, e.g., GB42053, glucose dehydrogenase GB51814, alpha glucosidase GB54549, alpha amylase GB49854, cytochrome P-450 9e2 GB43713, two trypsins GB41097, a two chymotrypsin inhibitors GB45614, GB50116, a methyltransferase GB44871, and a putative multivitamin transporter GB48560 were all elevated in R bees injected with virus.
